# Near-atomic structure of Japanese encephalitis virus reveals critical determinants of virulence and stability

**DOI:** 10.1038/s41467-017-00024-6

**Published:** 2017-04-26

**Authors:** Xiangxi Wang, Shi-Hua Li, Ling Zhu, Qing-Gong Nian, Shuai Yuan, Qiang Gao, Zhongyu Hu, Qing Ye, Xiao-Feng Li, Dong-Yang Xie, Neil Shaw, Junzhi Wang, Thomas S. Walter, Juha T. Huiskonen, Elizabeth E. Fry, Cheng-Feng Qin, David I. Stuart, Zihe Rao

**Affiliations:** 1 National Laboratory of Macromolecules, Institute of Biophysics, Chinese Academy of Science, Beijing, 100101 China; 2 Department of Virology, Beijing Institute of Microbiology and Epidemiology, Beijing, 100071 China; 3 Division of Structural Biology, University of Oxford, The Henry Wellcome Building for Genomic Medicine, Headington, Oxford, OX3 7BN UK; 4 Sinovac Biotech Co., Ltd., Beijing, 100085 China; 5 National Institutes for Food and Drug Control, No. 2, TiantanXili, Beijing, 100050 China; 6 State Key Laboratory of Pathogen and Biosecurity, Beijing, 100071 China; 7 Anhui Medical University, Hefei, 230032 China; 8 Diamond Light Sources, Harwell Science and Innovation Campus, Didcot, OX11 0DE UK; 9 Laboratory of Structural Biology, School of Medicine, Tsinghua University, Beijing, 100084 China

## Abstract

Although several different flaviviruses may cause encephalitis, Japanese encephalitis virus is the most significant, being responsible for thousands of deaths each year in Asia. The structural and molecular basis of this encephalitis is not fully understood. Here, we report the cryo-electron microscopy structure of mature Japanese encephalitis virus at near-atomic resolution, which reveals an unusual “hole” on the surface, surrounded by five encephalitic-specific motifs implicated in receptor binding. Glu138 of E, which is highly conserved in encephalitic flaviviruses, maps onto one of these motifs and is essential for binding to neuroblastoma cells, with the E138K mutation abrogating the neurovirulence and neuroinvasiveness of Japanese encephalitis virus in mice. We also identify structural elements modulating viral stability, notably Gln264 of E, which, when replaced by His264 strengthens a hydrogen-bonding network, leading to a more stable virus. These studies unveil determinants of neurovirulence and stability in Japanese encephalitis virus, opening up new avenues for therapeutic interventions against neurotropic flaviviruses.

## Abstract (web summary)

Japanese encephalitis virus (JEV) is a Flavivirus responsible for thousands of deaths every year for which there are no specific anti-virals. Here, Wang *et al*. report the cryo-EM structure of mature JEV at near-atomic resolution and identify structural elements that modulate stability and virulence.

## Introduction

Flaviviruses are positive-stranded RNA viruses that are mainly transmitted by mosquitoes and ticks. Many different flaviviruses have been identified and well-known examples include West Nile virus (WNV), Japanese encephalitis virus (JEV), dengue virus (DENV), yellow fever virus and Zika virus (ZIKV)^1^. These emerging or re-emerging flaviviruses have caused explosive epidemics around the world, as reported with the current ZIKV outbreaks in the America^2^ and the spread of JEV and WNV into new geographic areas over the last few years^3, 4^, which has caused considerable concern over public health safety. Although all flaviviruses are serologically related, they can be divided into a number of distinct virus groups. JEV, WNV, St Louis encephalitis virus (SLEV) and Murray Valley encephalitis virus, belonging to the JEV group have been consistently associated with human cases of encephalitis. Worldwide, some 50,000–175,000 clinical cases of Japanese encephalitis are reported in more than 25 countries annually^5^. Up to now no efficient antiviral therapy has been available. There are multiple vaccines for JEV, but they are not universally available due to cost, safety concerns, and licensing issues^6, 7^.

To cause encephalitis, the virus must gain entry to the central nervous system (CNS), a process known as viral neuroinvasiveness, and must replicate and cause damage within the CNS, a phenomenon known as neurovirulence. JEV strain P3 is highly neurovirulent and neuroinvasive in mice (the most virulent JEV strain known), whereas the JEV strain SA14 is relatively “weakly” virulent^8^ and a cell culture-derived JEV strain SA14-14-2 (derived from the SA14 wild-type strain) is further attenuated^9^. Over the past 20 years, many investigators have sought to discover the major genetic determinants critical for the virulence of JEV and identified a number of residues in the envelope (E) protein and some non-structural proteins, which significantly attenuate pathogenic JEV isolates^10–13^. However, the molecular mechanisms underlying virulence are not clearly understood.

During the viral life cycle, flaviviruses undergo a series of conformational changes from the immature to the mature, fusogenic, form of the virion^14^. In the endoplasmic reticulum of an infected cell, newly assembled immature flaviviruses have a rough surface with 60 spikes, each composed of E trimers with associated prM (E and M are envelope glycoproteins)^15^. Low pH triggers rearrangement of the E proteins into dimers and prM is cleaved by the protease furin in the *trans*-Golgi network^14^. However, E maintains interactions with pr and M until it encounters the neutral pH of the extracellular environment, where pr is released to yield the smooth mature virion^14^. Mature virions attach to cells and are taken into the endosome where the acidic environment leads to formation of an irreversible fusogenic E trimeric form^16^, exposing the fusion loops which engage the endosomal membrane leading to fusion of host and viral membranes and release of the viral nucleocapsid into the cell. Although low resolution cryo-electron microscopy (cryo-EM) structures of mature and immature WNV, recent near atomic resolution cryo-EM structures of DENV2, ZIKV and ex situ crystal structures of some domains of E have been reported^17–23^, there is no in situ structural information at high resolution available for encephalitic flaviviruses.

We set out to clarify the mechanism for neurovirulence and to gain an understanding of the pathogenesis of encephalitic flaviviruses in the JE sero-group using a combination of structural analysis, cellular assays, reverse genetics, and animal studies of mouse models. Here we report the cryo-EM structure of JEV strain P3 in its native form at near-atomic resolution. The structure reveals unusual “holes” between two monomers within a dimer, formed by cooperative shifts of the k-l hairpin, and i-j, E_0-_F_0_ and B_0-_C_0_ loops. These holes are surrounded by five encephalitis-specific motifs, which have been proposed to play a key role in the attachment of the encephalitic flaviviruses to their receptor(s). We have identified features that impart structural stability to the virus and thus play a role during the conformational changes of JEV. Notably, the Q264H mutation observed in an attenuated strain of JEV increases virus stability, hindering the series of conformational changes in the virion required for infection. Our studies on this attenuated strain of JEV further identify amino acids that impart neurovirulence in mice. The probable mechanism of attenuation of neurovirulence and its therapeutic implications are discussed.

## Results

### Structure determination

JEV (P3 strain) was cultivated in Vero cells at 37 °C and purified using a standard protocol (see Methods). Cryo-EM micrographs of the purified virions were recorded using a Polara electron microscope (FEI, Hillsboro, OR) equipped with a Gatan K2 Summit detector. Similar to the Cryo-EM studies on DENV2^24^ spherical (mature), almost spherical with local flaws (partially immature) and irregular (broken or fusion conformation) JEV particles were present (Supplementary Fig. 1). A total of 15,018 “perfect” particles were subjected to three-dimensional (3D) reconstruction. A final resolution of 4.3 Å (the ‘‘gold’’ standard Fourier shell correlation = 0.143) was achieved. The backbone of the polypeptide as well as many bulky side chains were clearly defined, allowing an atomic model for E and M to be manually built in (Supplementary Fig. 1). The model was refined and validated using standard X-ray crystallographic metrics (Supplementary Table 1).

### Overall structure and structural comparisons

**Fig. 1:**
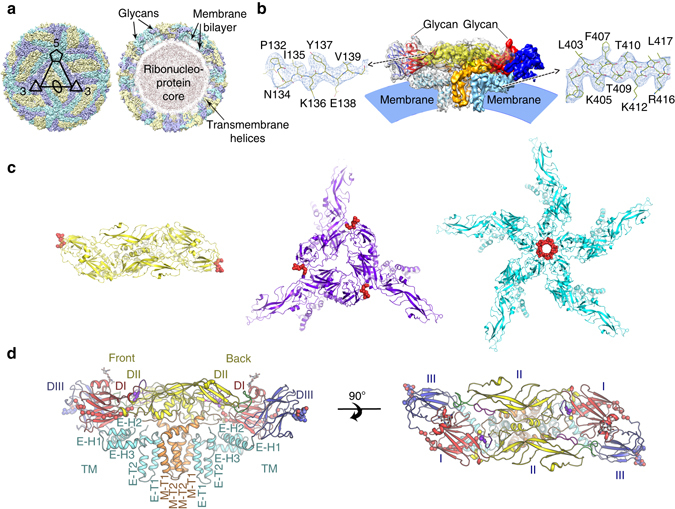
Overview of the cryo-EM structure of JEV (P3 strain). **a** 3D reconstruction (*left*) and a thin slice of the central section (*right*) of JEV viewed down an icosahedral twofold axis. E:M heterodimers of the same color are related by icosahedral symmetry. Heterodimers of different colors are quasi-equivalent, with *cyan* E:M dimers falling on the icosahedral fivefold axes, *blue* on the threefold, and *yellow* on the twofold. **b** Side view of the averaged heterodimer. Electron density maps for representative areas are shown. **c** RGD motifs locations. RGD motifs are represented as *spheres* in *red* for views of the twofold (*left*), threefold (*middle*), and fivefold (*right*), respectively. **d** Side view of the atomic model of the E:M:M:E heterotetramer shown in *ribbon*. Domain I, II, III, transmembrane (TM) of E and M are colored in *red*, *yellow*, *blue*, *cyan*, and *orange*, respectively. Glycans at Asn154 and RGD motifs are shown as *sticks* and *spheres*, respectively. E residues 279 to 297, rich in positively charged residues, are presented as *small balls*. The fusion peptide, i-j and k-l loops are highlighted in *green*, *magenta*, and *purple*, respectively

**Fig. 2:**
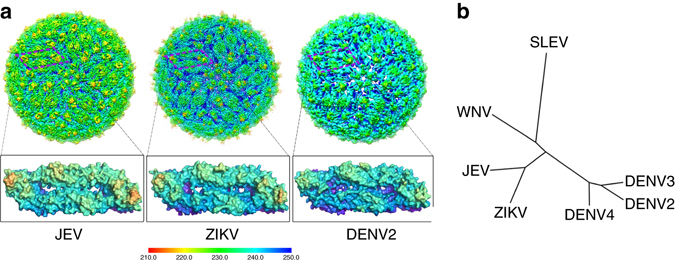
Structural comparisons of JEV with other flaviviruses. **a** Comparison of cryo-EM maps from JEV (P3 strain), ZIKV^23^ and DENV2^24^. The maps are colored by radius from *red* to *blue* according to the scale bar shown. The antiparallel dimers (E:M:M:E) marked by *purple squares* are highlighted. **b** Structure-based phylogenetic trees of representative flaviviruses: WNV, West Nile virus [PDB code: 2HG0]^19^; SLEV, St Louis encephalitis virus [PDB code: 4FG0]^57^; DENV, Dengue virus, DENV2 [PDB code: 3J27]^24^, DENV3 [PDB code: 1UZG]^58^, DENV4 [PDB code: 3UAJ]^59^; ZIKV, Zika virus, [PDB code: 5IRE]^22^; JEV, Japanese encephalitis virus. Note: DENV2, ZIKV, and JEV are from cryo-EM structures; WNV, DENV3, DENV4 are pre-fusion conformations from a crystal structure, SLEV is a post-fusion conformation from a crystal structure

The cryo-EM structure of mature JEV (P3 strain) reveals an icosahedral virion of ~ 510 Å in diameter, slightly bigger than DENV2 and ZIKV (~ 500 Å) (Figs. 1 and 2a). There are 180 copies of each of the E and M proteins per virion with each icosahedral asymmetric unit containing three copies each of E and M (Fig. 1). Three E-M-M-E heterodimers lying parallel to each other form a raft and 30 such rafts cover the viral surface. The internal capsid of JEV is disordered, as was the case in the Cryo-EM structures of DENV2 and ZIKV^21–24^. The E proteins form the outer shell and are anchored to the lipid bilayer envelope through their transmembrane helices, together with the M protein (Fig. 1b). On the surface of the virion, Asn 154 of each E protein is modified by high mannose glycosylation (Fig. 1b and Supplementary Fig. 2a), and these sugars are putative-binding sites for the cellular receptor DC-SIGN^25^. The RGD motif in domain III is exposed on the external surface both around the 5- and 3- fold icosahedral axes and centrally between the 5- and 3-fold axes (Fig. 1c and Supplementary Fig. 2b), and may act as points of attachment to cellular integrins. A *circa* 20 amino-acid stretch of E (residues 279 to 297, corresponding to *β* strand I_0_ and the linker between domain I and III), rich in basic residues and conserved across the DENV and JEV serological complexes, has been proposed to bind glycosaminoglycans (GAGs)^26^. Surprisingly, we find that most of the residues implicated in binding GAGs are buried, except for 279 and 297 K, suggesting that major conformational rearrangements would be required to make these amino acids accessible for binding GAGs (Fig. 1d and Supplementary Fig. 2c).

The in situ structure of JEV E consists of five domains, of which domains I, II, and III (DI, DII, DIII) make up the ectodomain. A superposition of our JEV (strain P3) cryo-EM structure onto the DENV2^24^, ZIKV^22^ cryo-EM structures and the crystal structures of WNV^19^ and JEV^20^ (SA14-14-2) E proteins shows that the structures are highly similar (Supplementary Figs. 3 and 4). As expected, the ectodomain of JEV strain P3 most closely resembles that of attenuated strain SA14-14-2 with an r.m.s. deviation of 1.3 Å, but a number of loops with the attenuated-specific mutations differ in conformation, e.g., the glycan loop in DI, the fusion loop, the i-j and k-l loops in DII, and several loops in DIII (Supplementary Fig. 4). 3D structure-based evolution analysis is well established^27^ and has been shown to be more robust than to sequence-based analyses where the sequence similarity is limited, since the structure directly determines biological functions^28^. Given that the sequence similarities of the flaviviruses from different groups are relatively low (<40%), we performed a structure-based phylogeny, which suggests that encephalitic flaviruses (JEV group) are more structurally similar than are dengue viruses (DENV group), interestingly, ZIKV links the JEV and DENV groups and is more similar to the JEV group (Fig. 2b).

### Structural features underpinning the stability of mature JEV

**Fig. 3:**
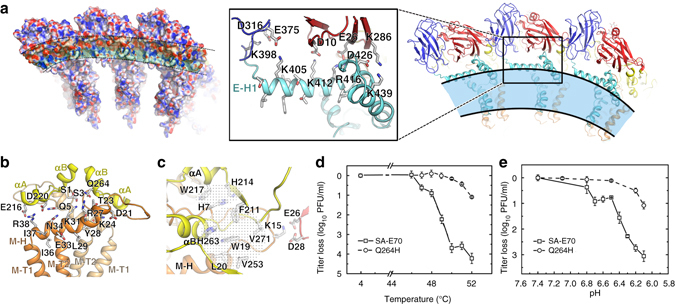
Structural elements modulating the stability of JEV. **a** Side view of the electrostatic surface (*left*) and the cartoon model (*right*) of the raft. Charge interactions between E-stem and E ectodomain are shown in the *zoom box*. The color scheme is the same as Fig. 1c. **b** Interactions between M-H and domain II. **c** M_1–20_ interacts with domain I and domain II. The *sticks* represent atomic models of selected side chains. Hydrophobic interactions are shown as a *dotted network*. All the structural representations shown in **a**–**c** are for JEV (P3 strain). **d** Loss of virus titer after heat treatment for 20 min at the indicated temperature. The treatment at 4 °C acts as the positive control. Q264H was constructed based on the infection clone of SA-E70 and contained a single amino-acid mutation in E protein. **e** Loss of virus titer after acid treatment for 20 min at the indicated pH values. Samples were tested in triplicate at each point, and the data were log10 transformed prior to calculation. Error bars show standard deviations

The helical stem (three perimembrane helices, E-H1, E-H2, and E-H3) and transmembrane region (E-T1 and E-T2) form the membrane anchor (Fig. 1d). The M protein contains an N-terminal loop followed by an amphipathic perimembrane helix (M-H) and transmembrane region (M-T1 and M-T2) (Fig. 1d). Interactions within the E:M:M:E heterotetramers and between them (through E to E interactions) contribute to the assembly of the virion and confer structural stability, as suggested by previous reports^29^. We observe that the inner surface of the E ectodomain, towards the E-H1 helix, is rich in negatively charged residues, while the amphipathic perimembrane helix E-H1 is highly positively charged (Fig. 3a). Charge interactions formed by Asp10, Glu26, Asp316, Glu375, Lys286, and Lys398 from the ectodomain and Lys405, Lys412, Arg416, Asp426, and Lys439 from the helical stem, which are highly conserved between all flaviviruses, link DI, DIII and the helical stem regions together (Fig. 3a). Similar to E-H1, M-H is also involved in interactions with DII and the N-terminus of a neighboring M through hydrogen bonds involving Glu216 and Asp220 from DII, and Ser3, Gln5, Lys31, Asn34, and Arg38 from M (Fig. 3b). There is a notable hydrogen-bonding network centered on Gln264 near helix αB. It appears that replacement of Gln264 with a Histidine in the attenuated phenotype of JEV could further strengthen this hydrogen-bonding network (Fig. 3b and Supplementary Fig. 5). The N-terminus of M (amino acids 1–20; M_1–20_) also participates in interactions with DI and DII (Fig. 3c). Lys15 from M_1–20_ interacts with Glu26 and Asp28 from DI through electrostatic interactions (Fig. 3c). Interestingly, all three residues are highly conserved in encephalitic flaviviruses. Trp19 and Leu20 of M_1–20_ form a hydrophobic core with residues of DII including Phe211, Val253, His263, and Val271, while His7 of M_1–20_ together with residues His214 and Trp217 from DII constitute a second hydrophobic core (Fig. 3c). These charged and hydrophobic interactions result in the stability of mature JEV.

### Amino acid 264 of E modulates particle stability

The attenuated SA14-14-2 strain of JEV shares ~ 99% amino-acid sequence similarity in the E protein but harbors a Q264H mutation compared to wild-type strains SA14 and P3 (Supplementary Table 2). A possible structural consequence this was noted above—replacement of Gln264 with a histidine might enhance the surrounding hydrogen-bonding network, leading to increased stability (Fig. 3b and Supplementary Fig. 5). In support of this, we find that SA14-14-2 shows enhanced resistance to heat and acid compared to both wild-type strains (Supplementary Fig. 6). To further test this hypothesis, a single Q264H mutation was introduced into the E portion of the full-length infectious cDNA of SA-E70 (a chimeric JEV where the prM-E gene of SA14-14-2 is replaced with that of SA14), and the stability of the rescued mutant Q264H and the parental virus SA-E70 determined. The titer of SA-E70 plummeted when exposed to temperatures above 48 °C. Thus, the titer of wild-type virus decreased more than 10,000-fold at 52 °C, while that of Q264H mutant decreased only tenfold at the same temperature (Fig. 3d). Consistent with the results of heat treatment experiments, SA-E70 suffered a steep loss in titer at pHs below 6.4 (1000-fold at pH 6.1). The Q264H mutant was more stable and under identical conditions experienced only a tenfold loss in titer (Fig. 3e). We also introduced a number of other mutations at the same position and tested the thermal stability of these mutants. Replacement of Gln264 with either alanine, arginine, asparagine, lysine, or threonine resulted in a dramatic decrease in viral titers upon heating to 48 or 50 °C (Supplementary Fig. 5). This rapid decrease in viral titers could be due to the formation of fewer hydrogen bonds because of either smaller side chains of the substituted amino acids or due to charge repulsion between the substituted amino acid and surrounding positively charged residues such as R27, and K31 of M. Thus, our data demonstrate that a single amino acid at position of 264 of E protein near helix αB plays a critical role in modulating the stability of JEV.

### Notable “holes” on the surface of the JEV

**Fig. 4:**
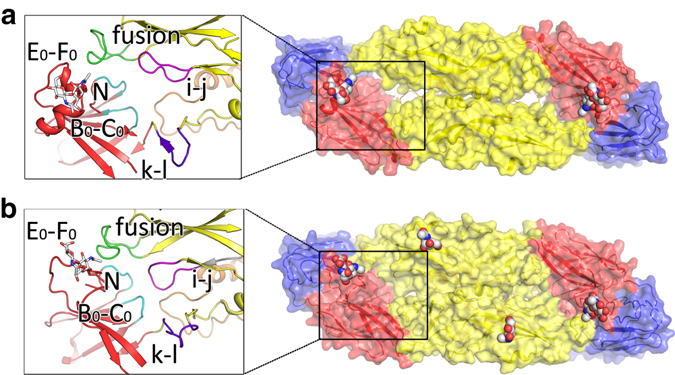
The “hole” on the JEV surface. The surface representations of E:M:M:E heterodimers from JEV(P3 strain) **a** and DENV2 **b**. Glycans are shown as *spheres* and *sticks* in the surface representations and in the cartoon representations, respectively. Domain I, II, III of E and M are colored in *red*, *yellow*, *blue*, and *orange*, respectively. The N-terminal loop of the A_0_ strand (labeled as N) and the B_0_-C_0_ loop are colored in *cyan*

The most unusual feature of the mature JEV (P3 strain) is the presence of notable holes between the two E:M units that exist as an antiparallel dimer (E:M:M:E) (Figs. 2a and 4a), although smaller “holes” were also observed in DENV previously^24, 30^ (Fig. 4b). In the structure of DENV2^24^, there are extensive interactions, hydrophobic as well as hydrophilic, between monomers within a dimer. These interactions are clustered in two regions. The first region is around the i-j loop and the fusion loop, which interact with the N-terminal loop of the A_0_ strand, the B_0_-C_0_, E_0_-F_0_ loops in DI and the k-l loop in DII from the opposite monomer (Fig. 4b). The second region of contact between the monomers in the dimer occurs around helix αB in DII, with interactions across the dyad axis. In JEV (P3 strain) the N-terminal loop of the A_0_ strand and the B_0_-C_0_, E_0_-F_0_ and k-l loops all recede slightly, which combined with conformational changes in the i-j loop, conspire to form a notable hole in the center, with part of M_1–20_ underneath (Fig. 4a). In addition αB-αB interactions are roughly halved compared with those in the DENV2 so that, overall, JEV (P3 strain) exhibits markedly fewer interactions between monomers within the dimer (this is also observed in the crystal structure of JEV E ectodomain)^20^, which may account for the multiple particle morphologies we observe (Supplementary Fig. 1a). Histidine residues that act as pH sensors in flaviviruses to mediate viral conformational changes have been studied extensively^31–34^. In the DENV group, His244 (His246 in JEV, strictly conserved in all flaviviruses) and His27 (conserved in the DENV group), might act as pH sensors, with protonation causing electrostatic repulsion at low pH, triggering a cascade of conformational changes to open the “hole” while deprotonation might allow hydrophobic and electrostatic interactions to come into play to close the “hole” (Supplementary Fig. 7). However, JEV (P3 strain) failed to close the “hole” under neutral pH conditions, perhaps because a number of residues highly conserved in the JEV group, including Asp28, Arg44, Lys166, Glu243, Glu244, Glu273, and Lys279 may, together with His264, produce enough electrostatic repulsion to maintain an open conformation at both low and neutral pH (Supplementary Fig. 7). These residues bear different or opposite characteristics in other flaviviruses (Supplementary Fig. 8).

### Binding sites for putative receptor(s) of the JEV group

**Fig. 5:**
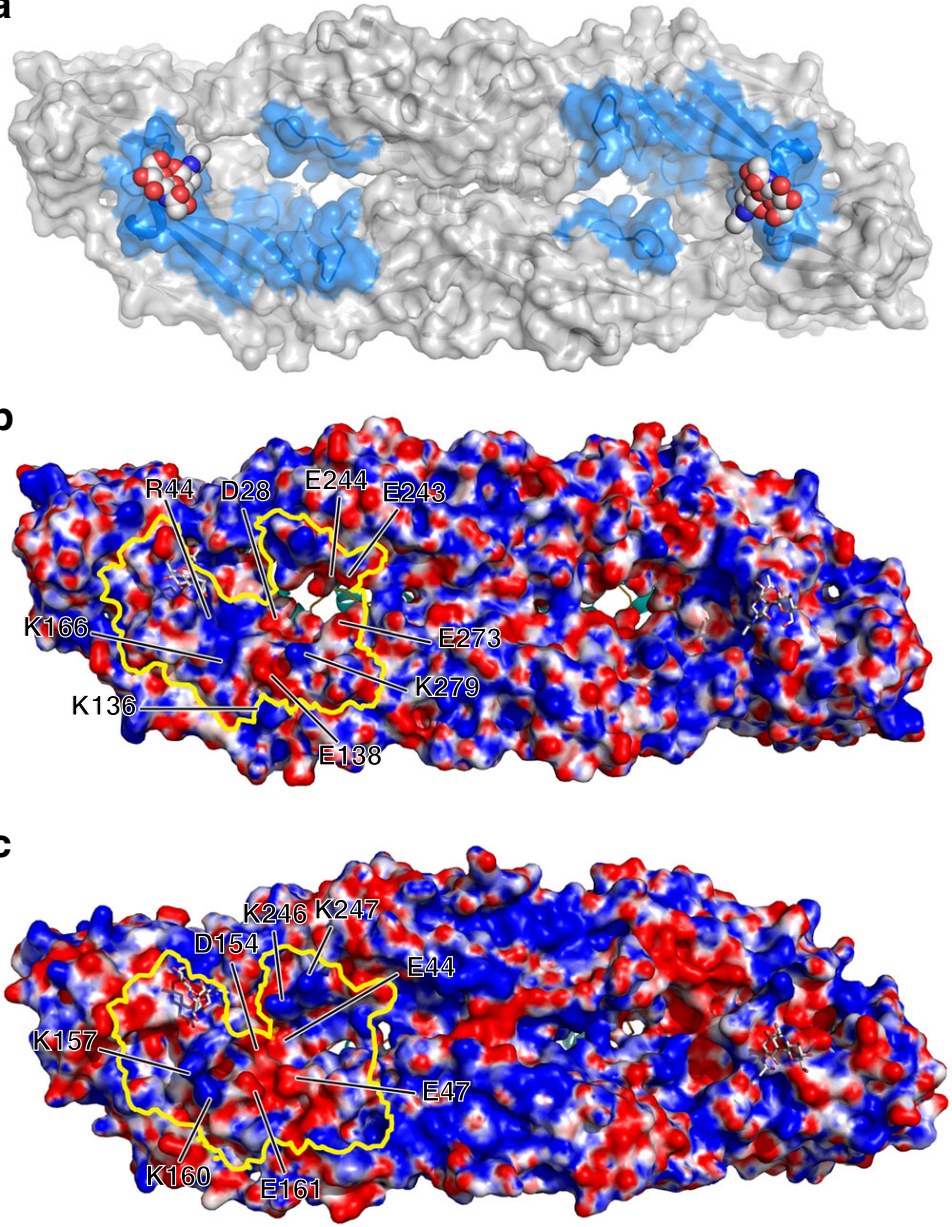
Putative receptor(s)-binding sites. **a** Location of encephalitis-specific motifs on the external surface of JEV (P3 strain) E:M:M:E heterodimers. The encephalitis-specific motifs are highlighted in marine. Glycans are shown as *spheres*. **b** External electrostatic surface of JEV E:M:M:E heterodimers and **c** DV2E:M:M:E heterodimers. The encephalitis-specific motifs are outlined in *bright yellow*. The residues determining the electrostatic features in the encephalitis-specific motifs are marked

The entry of flaviviruses into their target cells is mediated by the interaction of E with cell surface receptors. The JEV group may infect neurons by using receptor(s) distinct from those used by other flaviviruses^35^. The site for the attachment of such neuronal receptor(s) will presumably be conserved in the JEV group. Structure-based sequence alignment of the E proteins from seven representative flaviviruses revealed seven encephalitic-specific motifs (Supplementary Fig. 8), five of which are located on the external surface surrounding the “holes” (Fig. 5a), which might provide clues to analyze features common to the JEV group and different to other flaviviruses. Of note, the structures of motif 3 (residue 137–168) are the most divergent between JEV and DENV2 and ZIKV (Supplementary Fig. 4). Furthermore, this region exhibits a distinct electrostatic surface between JEV (P3 strain) and DENV2, largely due to residues Asp28, Arg44, Lys136, Glu138, Lys166, Glu243, Glu244, Glu273, and Lys279 of JEV and residues Glu44, Glu136, Asp154, Lys157, Lys160, Glu161, and Lys246 of DENV2 (Fig. 5b,c). Of these residues, Arg44, Glu138, Glu243, and Glu244 are highly conserved in the JEV group, whereas their counterparts in other flaviviruses are residues with opposite charge. In addition mutations E138K, E244G, and K279M were observed in the SA14-14-2 (Supplementary Table 2) and were also reported to be involved in neurovirulence in mice^11^. Interestingly, residue Glu244, within the ij loop, locates at the bottom of the “holes”, the E244 mutations (except E244D) exhibit growth defects and decrease JEV infectivity in neuronal cells^11^. The mutation E244G might partially contribute to the attenuation of SA14 compared to the P3 strain. We, therefore, propose that these JEV group-specific motifs with distinct electrostatic features might attach to a common receptor for the JEV group and that residue Glu138 may play a critical role in receptor binding.

### Glu138 plays an important role in conferring neurovirulence

To verify the roles of the E in the virulence of JEV, we replaced the prM-E gene of the attenuated SA14-14-2 strain with those of the wild-type virus SA14. This chimeric JEV was named SA-E70 (Supplementary Fig. 9a). Following recovery in BHK-21 cells, all three types of viruses formed homologous plaques in BHK-21 cells (Supplementary Fig. 9b). SA-E70 replicated as efficiently as SA14 and SA14-14-2 in BHK21, Vero and HepG2 cells (Supplementary Fig. 9c).The virulence phenotype of SA-E70 was similar to SA14 in adult mice (Supplementary Fig. 9d), suggesting that the prM-E gene harbors the mutations responsible for the difference in virulence between SA14 and SA14-14-2. Since no mutations were identified in the *prM* gene, a subset of the eight amino-acid substitutions in the E protein are likely to be responsible for the attenuation phenotype.

**Fig. 6:**
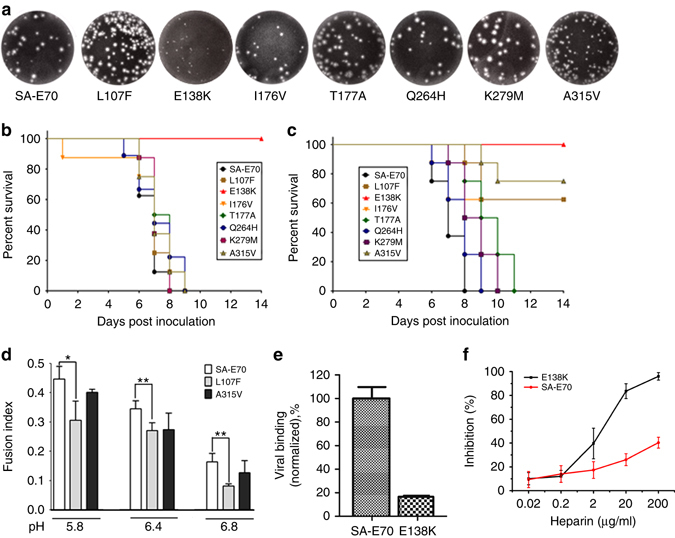
A single amino acid, Glu138, determines JEV neurovirulence. **a** Plaque phenotypes of the mutant viruses. Survival curve of 3-week-old BALB/c mice infected with the indicated viruses by i.c. (C, 80 PFU/mouse) **b** or i.p. route (D, 5 × 10^7^ PFU/mouse) **c**. **d** Percent fusion of mutant viruses relative to SA-E70in C6/36 cells. Triplicate wells of mock infected cells and SA-E70 infected controls were included in each assay. Asterisks represent significance: *P<0.05 and **P<0.01. **e** Binding affinity comparison between SA-E70 and E138K mutant virus to SH-SY5Y cells. The amount of virus on the cell surface was estimated by RT-PCR, after three washes, when exposed to SH-SY5Y cells at 4 °C for 30 min. **f** Inhibition by heparin of virus infectivity in Vero. SA-E70 and SA-E70 E138K mutant virus were incubated with heparin (0.02, 0.2, 2, 20, 200 μg/ml) prior to addition to cells. Agar overlay was added to allow plaque formation after 1 h of adsorption at 37 °C. Inhibition rate was calculated as [no. plaques (controls)—no. plaques (heparin treatment)/no. plaques (controls)]. Experiments were carried out in triplicate and error bars show standard deviations

To attempt to decipher the role of the specific amino-acid substitutions identified in the E protein of the attenuated SA14-14-2 strain, we introduced each of these mutations (except for K439R) individually into the chimeric SA-E70 virus (Supplementary Fig. 10a). All mutant viruses showed similar growth curves in BHK-21 cells (Supplementary Fig. 10b). Of all rescued viruses, E138K showed a unique small plaque phenotype (Fig. 6a). We then compared the neurovirulence and neuroinvasiveness of these mutant viruses with SA14 and SA14-14-2 in mice. As shown in Fig. 6b, the single mutation E138K completely attenuated neurovirulence in mice, while the others had no effect. For neuroinvasiveness, the E138K mutation resulted in complete attenuation, while the L107F and A315V mutations led to a partly attenuated phenotype (Fig. 6c). Taken together, our results indicate that the E138K mutation plays a pivotal role in producing the attenuated phenotype of SA14-14-2. In addition, L107F and A315V can cause partial attenuation.

To further clarify the molecular mechanism of attenuation, we first confirmed that there is no significant difference in viral protein expression among the three mutant viruses harboring E138K, L107F, and A315V (Supplementary Fig. 10c). Cell fusion assays demonstrated that L107F significantly decreased fusion ability while A315V slightly reduces fusion ability at low pH (Fig. 6d). In line with cell fusion results, Leu107 is located on the fusion loop and Ala315 contributes to the formation of the hydrophobic cavity where the fusion loop is buried (Fig. 1d). The replacement of Leu107 and Ala315 with Phe107 and Val315, respectively, seems to further stabilize the fusion loop. To verify the role of the residue Glu138 in receptor(s) binding, we performed cell-binding assays. The E138K mutant virus showed a significantly reduced binding ability to human neuroblastoma SH-SY5Y cells (Fig. 6e). Additionally, heparin inhibition assay showed that the E138K mutant virus was more susceptible to inhibition by heparin (GAGs) during infection of Vero cells (Fig. 6f).The enhanced affinity for GAGs, which are ubiquitously present on the cell surface and extracellular matrix, prevents viremia of sufficient magnitude for viral entry into the brain parenchyma^36^.

## Discussion

Flaviviruses are adapted to replicate in different hosts with different internal environments, for example, in humans at 37 °C and mosquitoes at ambient temperature. This adaptation may result in the virus structure being different when produced indifferent cell lines. Indeed it has been reported that the DENV2 structure differs at the temperatures of its human and mosquito hosts (smooth surface below 33 °C and bumpy appearance at 37 °C)^32^, while DENV1 and DENV4 maintain the same appearance (smooth particles)^37^. Although Shimojima, M. *et al*.^38^ found distinct usage of receptors by JEV in different cell lines, JEV particles produced from both Vero and C6/36 cells show a smooth appearance at 37 °C (Supplementary Fig. 11), consistent with the observation that structures for ZIKV prepared from mammalian and insect cells are indistinguishable^22, 23^. We have no simple explanation of why some flaviviruses undergo conformational transitions matching environmental (host or temperature) changes, while others do not. Further studies are needed to clarify the mechanism.

Our structure of mature JEV (P3 strain) provides near-atomic information on encephalitic flaviviruses, revealing unusual “holes” on the surface. During their life cycle, flaviviruses undergo a series of conformational changes. These changes are particularly important for host cell entry. The angle between DI and DII in E varies substantially throughout the viral life cycle. Rotation of DII would facilitate the conformational change of E required for the virus to start fusion with the endosomal membrane^39^. Superposition of in situ JEV DI and DII over homologous structures of E proteins using Dyndom^40^ reveals that the largest deviation (29.4°) is observed between positions of DII in JEV and the immature DENV2. This difference decreases to 18.3° in the mature form of DENV2 (Supplementary Fig. 12). We note that this is not only the largest change, but is also one that alters significantly during the viral life cycle. Furthermore, our structural results support the notion that M_1–20_ internalizes below the E dimer after furin cleavage of prM by passing through the “hole” and that this motion is mediated by highly conserved pH sensors, including His7 in M, His214, and His264 in E (Supplementary Fig. 7).

Gromowski and colleagues^10^ demonstrated 10 amino-acids substitutions (according to JEV SA14-14-2 vaccine-specific mutations) in the E protein which attenuated JEV India/78 strain. Several amino-acid residues in the E protein, including E-138^13^, E-244^11^, and E-279^12^, have also been reported to contribute to the virulence of JEV. These results suggest that the E protein is a principal determinant of virulence. We have identified five encephalitis-specific motifs in the E protein surrounding the “hole”, which probably mediate attachment to neurons *via* electrostatic interactions. Residue Glu138 of the E protein, which is part of motif 3, is one of the points of greatest divergence (in terms of both sequence and structure) between the encephalitic and non-encephalitic viruses and we demonstrate that it plays an essential role in the attachment of the virus to neural cells (Fig. 6e). Furthermore, substitution of Glu138 by a lysine resulted in the increased susceptibility of virus infectivity to inhibition with GAGs, which is consistent with the correlation between GAG-binding affinity and neurovirulence^36^. Indeed, tissue culture adaptation not in frequently involves the gain of GAG-binding sites, relaxing the requirement for the usual cell receptor interaction^41^. The best understood example of this structurally is foot-and-mouth disease virus^42^ and the simplest explanation of our results for JEV is that a key receptor for the encephalitic flaviviruses binds in the region we have identified, while mutation of Glu138 to a basic residue allows attachment of sulphated sugars of GAGs while simultaneously preventing cell entry via the usual receptor that confers neurovirulence. This is supported by the observation that the single E138K mutation is capable of completely blocking the lethal neurovirulence and neuroinvasiveness of JEV in mice. Furthermore Glu138 is highly conserved in the encephalitic flaviviruses (Supplementary Fig. 8), suggesting that, by targeting Glu138 and putative receptor(s)-binding sites, it may be possible to develop generic therapies against a number of encephalitic viruses posing serious threats to health.

## Methods

### Cells and viruses

BHK-21 (Baby hamster kidney), Vero (African green monkey kidney), and SH-SY5Y (Human neuroblastoma) cells were maintained in Dulbecco’s minimal essential medium (Invitrogen) supplemented with 10% fetal bovine serum and penicillin–streptomycin (100 μg/ml) at 37 °C. JEV vaccine strain SA14-14-2 (GenBank no. D90195), strain SA14 (GenBank no: D90194), and strain P3 (GenBank no. AY849939) were provided by the Chengdu Institute of Biological Products and National Institutes for Food and Drug Control, respectively. Virus titers were determined by standard plaque assay on BHK-21 cells, and virus stocks were stored in aliquots at −80°C.

### JEV reverse genetics

Full-length infectious clones of the parental and corresponding mutant viruses were constructed^43^, the plasmids linearized with *Xho*I and used as templates for SP6RNA polymerase transcription in the presence of m7GpppA cap analog. All rescued viruses were characterized by plaque forming assay, growth curve, indirect immunofluorescence assay, and full-genome sequencing. For details see Supplementary information.

### Particle production and purification

JEV virus genotype P3 was propagated in Vero cells at a multiplicity of infection (MOI) of 0.5 at 37 °C. The supernatant and cells were collected a week post infection, inactivated by incubation with formaldehyde (1:2000 dilution) at 4 °C for 7 days and centrifuged to remove cell debris. The supernatant was ultra-filtered using a 0.22 μm filter, concentrated with a 300 kD cutoff concentrator and then subjected onto a 30–55% (w/v) discontinuous sucrose gradient ultracentrifugation at 30,000 rpm for 12 h in an P35ZT rotor at 4 °C. Crude JEV concentrate (~ 0.6 mg in phosphate-buffered saline (PBS) buffer pH 7.0) was loaded onto a 15–45% (w/v) sucrose density gradient and centrifuged at 29,000 rpm for 3 h in an SW41 rotor at 4 °C. Fractions containing JEV were collected and dialyzed against PBS buffer.

To test whether JEV produced from insect cells exhibit a bumpy appearances, JEV (P3 strain) was propagated in C6/C36 cells at a MOI of 1 at 27 °C. The purification procedures for insect-produced JEV were similar to those for mammalian-produced JEV.

### Cryo-EM data collection

A 3 μl aliquot of purified JEV virions (P3 strain) (1 mg/ml) was applied to a freshly glow-discharged 400-mesh holey carbon-coated copper grid (C-flat, CF-2/1-2C,;Protochips Inc.). Grids were blotted for 3 s, in 90% relative humidity for plunge-freezing (Vitrobot; FEI) in liquid ethane. Cryo-EM data sets were collected at 300 kV with an FEITecnai G2 Polara microscope (FEI, Hillsboro, OR), equipped with a direct electron detector (K2 Summit; Gatan, Pleasanton, CA). Movies (25 frames, each 0.2 s, total dose 25 e Å^−2^) were recorded with a defocus between 1.0 and 2.5 μm in single electron counting mode using SerialEM^44^ at a calibrated magnification of 37,027×, resulting in a pixel size of 1.35 Å.

### Image analysis, model building, and refinement

Micrographs were corrected for beam-induced drift using MOTIONCORR^45^. A total of 30,558 good particles were selected by visual inspection from 1866 cryo-EM micrographs. Particles were picked manually using the boxer program in EMAN^46^ package. Contrast transfer function (CTF) parameters for each particle were estimated using Gctf^47^. Micrographs with signs of astigmatism or significant drift were discarded. The structure was determined using Relion 1.4^48^ with icosahedral symmetry applied. Two-dimensional (2D) alignment was performed in Relion. Non-circular classes were excluded iteratively from the 2D alignment until all classes were circular (Supplementary Fig. 2B). However, even good classes from 2D alignment still contained some “imperfect” particles (Supplementary Fig. 2C). The cryo-EM structure of DENV2 low-pass-filtered to 40 Å was used as an initial model for 3D classification and refinement. A total of 15,018 particles were used to obtain the final density maps at 4.3 Å, as evaluated by Fourier shell correction (threshold = 0.143 criterion), using gold-standard refinement. The crystal structure of the ectodomain of JEV (PDB code: 3P54)^20^ was initially fitted into the EM map with CHIMERA^49^ and further corrected manually by real-space refinement in COOT^50^. The models of E helical stem, transmembrane region, and M were built de novo into density with the structures of DENV2 as a guide using COOT, REFMAC^51^ was used to calculate the difference map that highlighted the areas where the model was incorrect. The model was further refined by positional and B-factor refinement in real space using Phenix^52^ and rebuilding in COOT iteratively. Only the coordinate was refined, the maps were kept constant. The final model was evaluated by Molprobity^53^ functions integrated in Phenix. The data set and refinement statistics are summarized in Supplementary Table 1.

### Virus stability assays

To test thermostability, viral stocks were incubated at the indicated temperature for 10 min, and remaining infectivity measured by plaque assay. Samples were tested in triplicate at each point, and the data were log_10_ transformed prior to calculation of averages and standard deviations. To test acid sensitivity, a modified version of a previously reported procedure^54^ was followed. Briefly, 100 mM phosphoric acid buffers at different pHs were mixed with viral solution followed by incubation at room temperature (~ 25 °C) for 10 min. After neutralization with 1 M Tris-HCl (pH 7.4), the remaining titer was determined by plaque assays on BHK-21 cells.

### Viral-binding assay

To assess the cell binding capability of JEV to SH-SY5Y, cells were incubated at 4 °C for 1 h with 10^5^ PFU of E70 and E138K, respectively^55^. Viral RNA copies from cell lysates after the remove of supernatant were determined by real-time PCR. Results are shown as mean ± SD from three independent experiments.

### Cell–cell fusion assay

To perform the cell–cell fusion assay^56^ C6/36 cells plated in a 24-well plate were infected with WT and mutant viruses at an MOI of 1 and then maintained in pH 7.7 culture medium at 28 °C for 3 days. Fusion was triggered at room temperature by the application of serum-free medium buffered between pH 5.8 and 6.8 for 2 h. The cells were fixed in 4% paraformaldehyde for 10 min at room temperature, and the nuclei stained with 4',6-diamidino-2-phenylindole for 10 min. Numbers of the total nuclei and the nuclei of the syncytia in a microscopic field were counted (at least five fields per well) for calculation of fusion index (FI), FI = 1−(number of cells/number of nuclei). Results were derived from two independent assays of each mutant virus.

### Indirect immunofluorescence assay

Confluent BHK-21 cells were infected with viruses at a MOI of 0.01. At the indicated time post infection, the cells were fixed with ice-cold acetone and incubated with the primary mouse monoclonal antibody (MAb) 4D5, specific for JEV E protein, followed by incubation with secondary goat anti-mouse IgG antibodies conjugated to Alexa Fluor 488 (Invitrogen). Positive cells were detected using fluorescence microscopy (Olympus).

### Heparin inhibition assay

Tenfold serial dilutions of heparin (ranging from 200 to 0.02 μg/ml) plus 200 PFU JEV mutant virus were pre-incubated at 37 °C for 1 h, and then incubated on confluent Vero cells in six-well plates at 4 °C for 2 h. The inoculums were removed and the cells washed three times with PBS to remove unbound virus. Cells were overlaid with 1% agarose-containing overlay medium and the plates incubated in a 5% CO_2_ incubator at 37 °C for 3 days for plaque formation. Cells inoculated without heparin were used as controls. Inhibition rate was calculated as [no. plaques (controls) −no. plaques (heparin treatment)/no. plaques (controls)].

### Mouse experiments

All animal experiments were conducted in strict accordance with the guidelines of the Experimental Animal Welfare and Ethics Committee of Beijing Institute of Microbiology and Epidemiology. A129 mice on the 129/SvEv genetic background were obtained from B&K Universal Ltd. and bred in a specific-pathogen-free facility.

For neurovirulence tests in 129 and A129 (129/SvEv mice deficient in IFN-*α*/*β*) mice, groups of 4-week-old 129 or A129mice (*n* ≥ 5) were intraperitoneally (i.p.) inoculated with 10^7^ PFU SA14-14-2, SA14 or SA-E-70, respectively. Mice were observed for 3 weeks after inoculation. Survival rate was derived from the number of mice that were killed at the moribund stage. Brains were collected immediately after killing of mice, homogenized, and dilute d with PBS to make the final 10 % (wt/vol) suspensions. After three cycles of freezing and thawing, these 10 % (w/v) tissue suspensions were centrifuged at 20,000×*g* at 4 °C for 1 h, virus titers in supernatant were determined by plaque assay on BHK-21 cells.

For neuroinvasiveness tests in BALB/c mice, groups of 3-week-old mice (*n* ≥ 5) were injected with 10^7^ PFU SA14, SA14-14-2 or the recombinant JE viruses via the i.p. route. The mortality was then monitored daily for 3 weeks. For neurovirulence tests in BALB/c mice, groups of 3-week-old mice (*n* ≥ 5) were intra cranially (i.c.) administered 80 PFU of the recombinant mutant JE viruses. The mice were monitored daily for 3 weeks to assess morbidity and mortality. Survival analyses were performed by log rank tests using GraphPad Prism software 5.0.

### Transcription and transfection

All the constructed plasmids were linearized with *Xho*I and used as templates for SP6RNA polymerase transcription in the presence of m7GpppA cap analog. In vitro transcription was done using the RiboMAX Large Scale Production System (Promega) according to the manufacture’s protocols. The integrity of transcripts was verified by 1% agarose gel electrophoresis. RNA transcripts from in vitro transcription were transfected into BHK-21 cells with Lipofectamine 2000 (Invitrogen), and the rescued viruses were harvested 3 to 4 days post transfection when typical cytopathic effects were observed. Working virus stocks were prepared by amplification of the transfection harvest for one passage in BHK-21 cells, and stored in aliquots at −80 °C until further use. Virus titers were determined by standard plaque assays using BHK-21 cells.

### Statistical analysis

For the survival analysis, the Kaplan–Meier survival curves were analyzed using a log-rank test and standard GraphPad Prism software 5.0. Average results were obtained from at least three independent experiments, and the Student’s test was used to assess the significant differences (*P* < 0.05).

### Data availability

Atomic coordinates have been submitted to the Protein Data Bank with accession number 5WSN. The cryo-EM density map of the virion has been deposited with the Electron Microscopy Data Bank EMD-6685. The additional data that support the findings of this study are available from the corresponding authors upon request.

## Electronic supplementary material

Supplementary Information Supplementary Figures and Supplementary Tables

Peer Review File Reviewer reports and authors' response from the peer review of this Article at Nature Communications
