# Supplementary material for: Near-atomic structure of Japanese encephalitis virus reveals critical determinants of virulence and stability
Source: Nat Commun. 2017 Apr 26;8:14. doi: 10.1038/s41467-017-00024-6 (PMC5432033; doi:10.1038/s41467-017-00024-6)
Supplement: Supplementary file 1 — Supplementary Figures and Supplementary Tables [file 41467_2017_24_MOESM1_ESM.pdf]

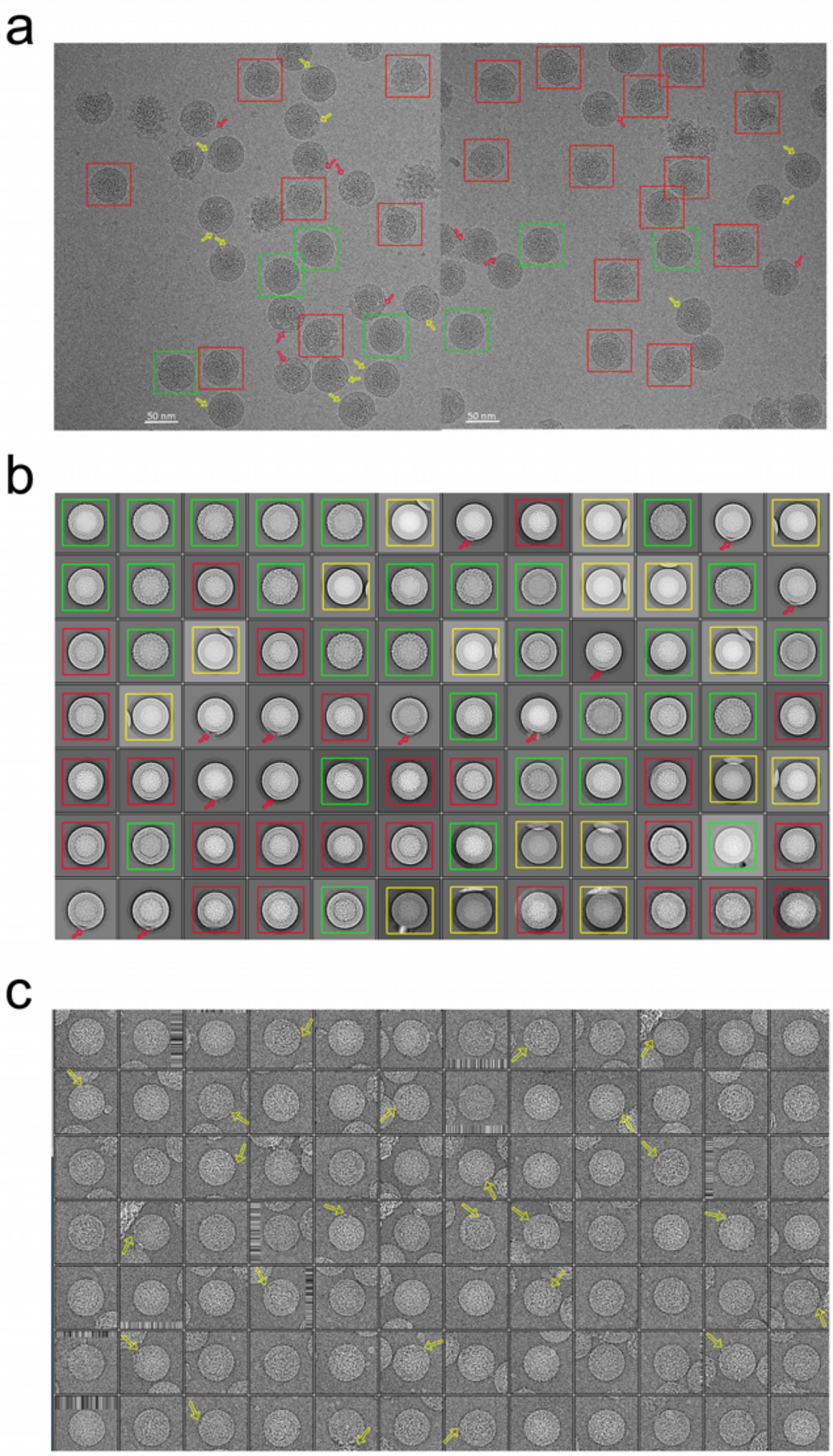
a
50 nm
50 nm
b
c

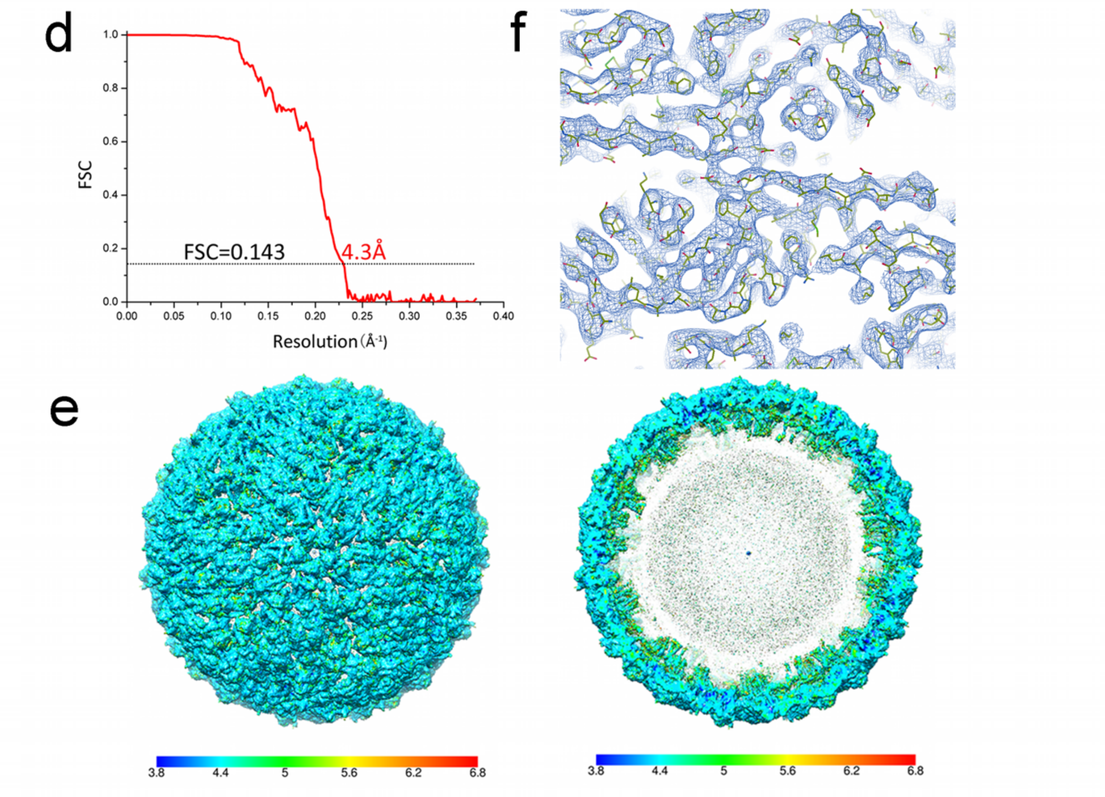

Supplementary Figure 1. Cryo-EM image, 2D classification and assessment of resolution.

(a) Cryo-EM image. Green and red boxed particles represent visually “good” particles and irregular particles, respectively. Particles appearing circular, but with a major or minor local deformation are marked with red or yellow arrow, respectively. **(b)** Reference-free 2D classification in Relion. Green, red and yellow boxed classes indicated relatively “good” classes, “useless” classes and “improper-alignment” classes, respectively. The classes with a notable local deformation were labeled with red arrows. **(c)** Particles from green boxed classes. The imperfect particles with a minor local deformation were labeled with yellow arrows. **(d)** The gold-standard FSC curves of the final maps. The resolution at FSC=0.143 is 4.3 Å. **(e)** The final JEV map

as analyzed by ResMap [1], showing a resolution distribution from 3.8 to 6.8 Å. Core parts of the map have resolution of ~4 Å. **(f)** Electron density map.

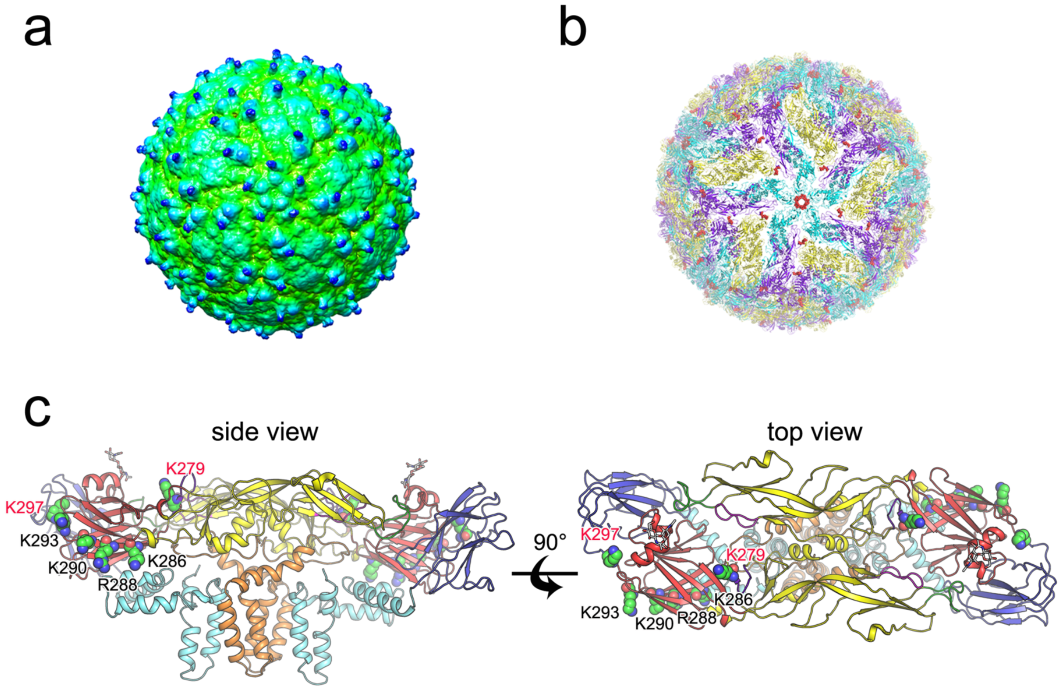

Supplementary Figure 2. Overall structure of JEV.

(a) Surface of the JEV cryo-EM map at lower contour level. The blue "protrusions" represent the glycans at Asn154. **(b)** Cartoon of the mature JEV virion looking down an icosahedral five-fold axis. The color scheme is same as that used for **Fig. 1d**. The RGD motifs are shown as small spheres on the surface. **(c)** A circa 20 amino acid stretch of E (residues 279 to 297), rich in basic residues. Side view and top view of the atomic model of the E:M:M:E heterotetramer shown in ribbon. The basic residues in the stretch of E (residues 279 to 297) are shown as spheres. The color scheme is same as that used for **Fig. 1d**.

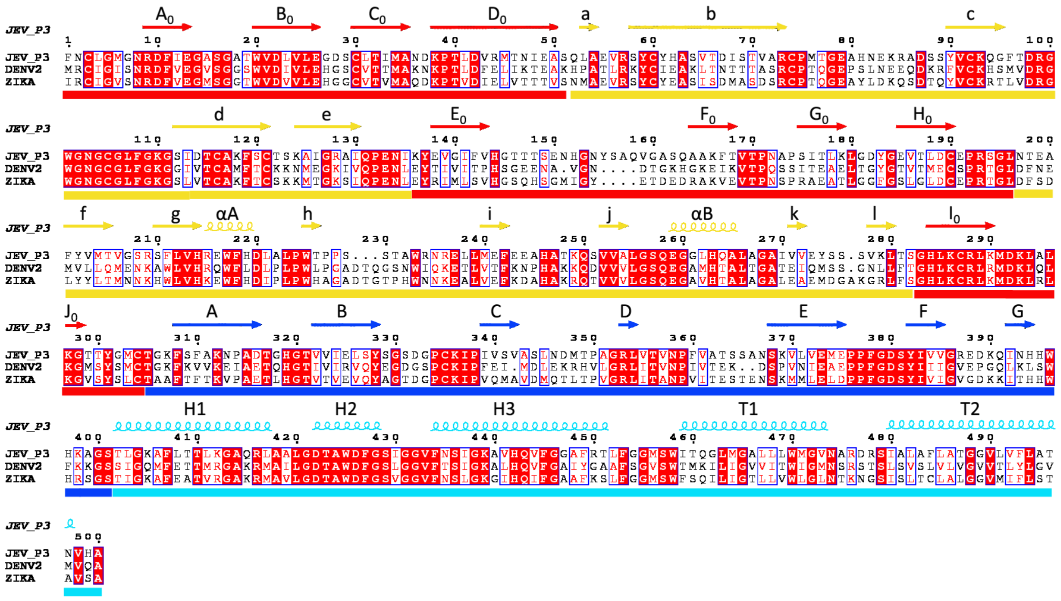

Supplementary Figure 3. Alignment of E protein sequences of JEV (P3 strain) with DENV2 and ZIKV.

DI, DII, DIII and stem (H1, H2, H3, T1 and T2) are indicated below the sequences and shown in red, yellow, blue, and cyan respectively. The secondary structures of JEV (P3 strain) were labeled above the sequences.

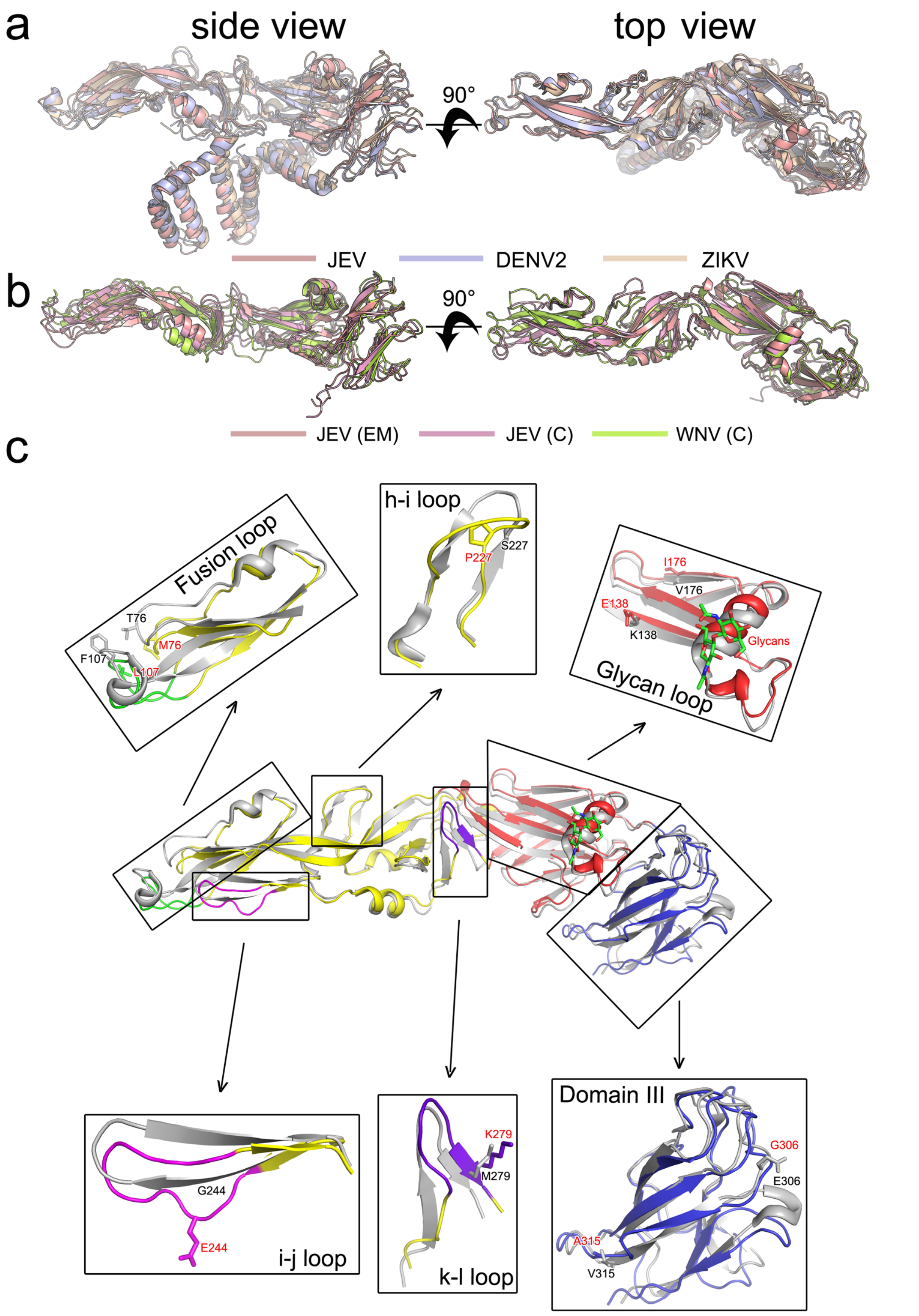

Supplementary Figure 4. Structural comparisons of JEV with DENV2, ZIKV and WNV.

(a) Superposition of E:M (envelope glycoproteins) monomer of JEV with that of DENV2[3] and ZIKV[2] (a key defines the colour for each virus) **(b)** Structural comparisons

of the E ectodomain between JEV (CM - cryo-EM structure (P3 strain)), JEV (C - crystal structure (SA14-14-2 strain))[4] and WNV (C - crystal structure)[5]. **(c)** Structural comparison of the E ectodomain between JEV P3 strain and JEV SA14-14-2 strain. The three domains and fusion peptide of the E monomer of JEV are colored as described in **Fig. 1d**. The comparisons of selected regions are enlarged and residues specifically mutated in SA14-14-2 strain are shown as sticks.

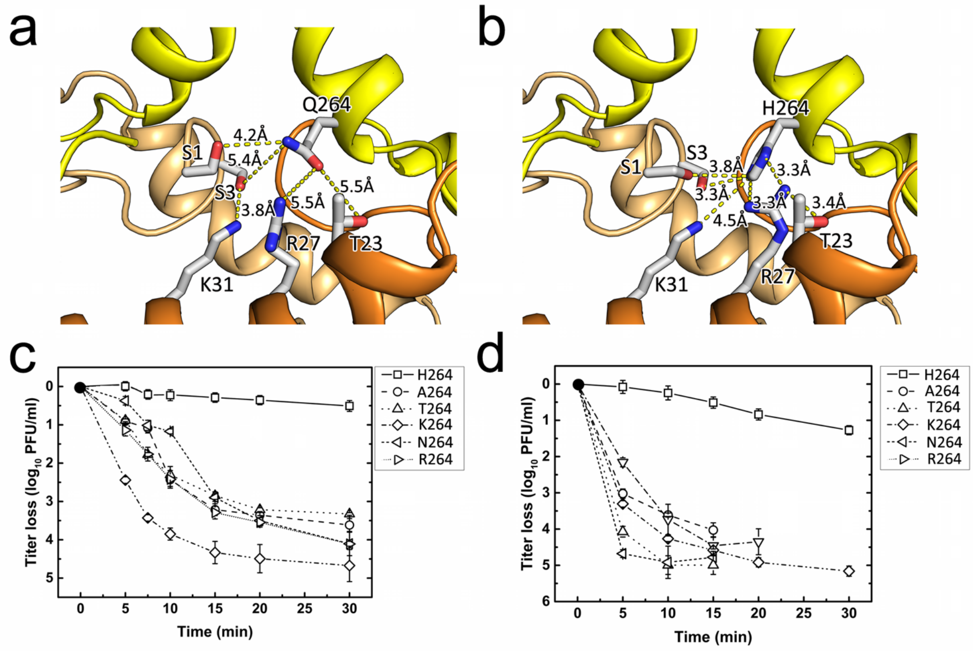

Supplementary Figure 5. A potential hydrogen-bonding network and the stability of JEV.

(a) A potential hydrogen-bonding network centering on Gln264 near helix αB is formed; **(b)** the same network after replacement of Gln264 with a histidine would be significantly strengthened. The residues involved in the formation of hydrogen bonds are shown as sticks. Evaluation of the stability of JEV mutants (mutation of E264 into different amino acids) by exposure to heat at 48 °C **(c)** or 50 °C **(d)** for the indicated time period. Reduction in titer of the viruses over a period of time was determined.

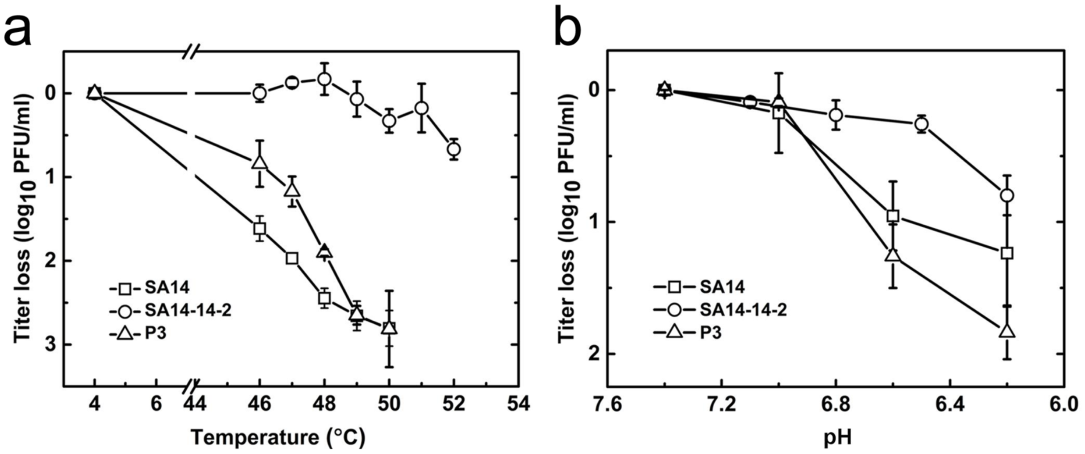

Supplementary Figure 6. Stability to heat and acids of wild type JEV strains.

Virus stability to heat (a) and acid (b) were assayed as previously described [6,7] and the remaining infectivity of each virus post treatment were measured by plaque assay on BHK-21 cells. Samples were tested in triplicate at each point, and the data were log10 transformed prior to calculation.

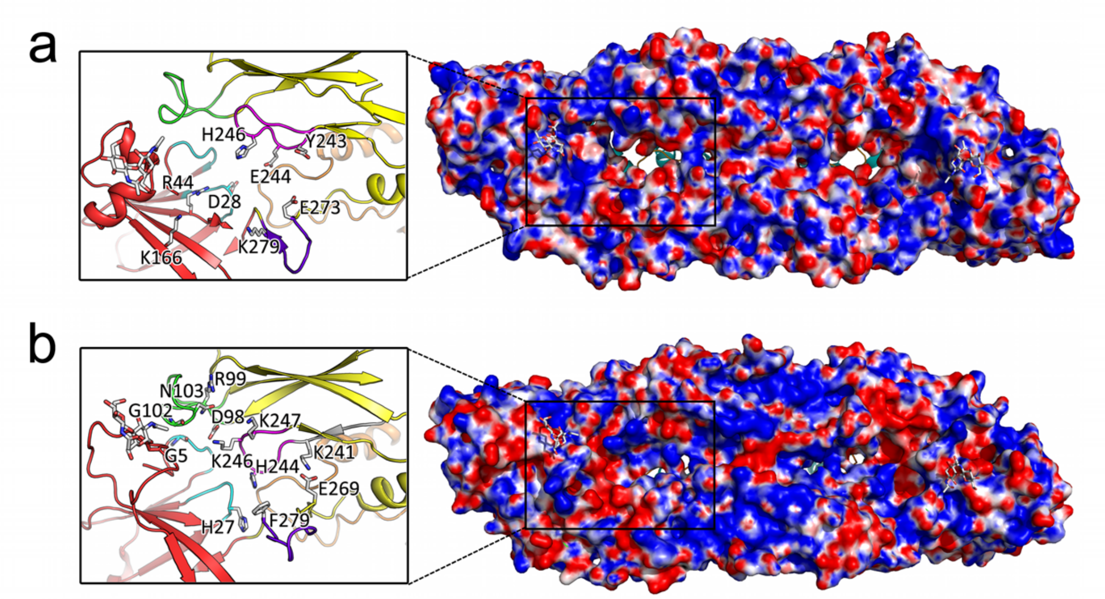

Supplementary Figure 7. Charge distribution on the surface.

External electrostatic surface charge distribution of the JEV E:M:M:E heterodimers **(a)** and the DENV2 E:M:M:E heterodimers **(b)**. The residues showing hydrophobic and hydrophilic interactions between the interface of the DEV2 and the residues involved in the formation of the "hole" on the surface of the JEV by electrostatic repulsion are represented as sticks. The color scheme is the same as that used in **Fig. 1d**. Glycans at Asn154 are also shown as sticks.

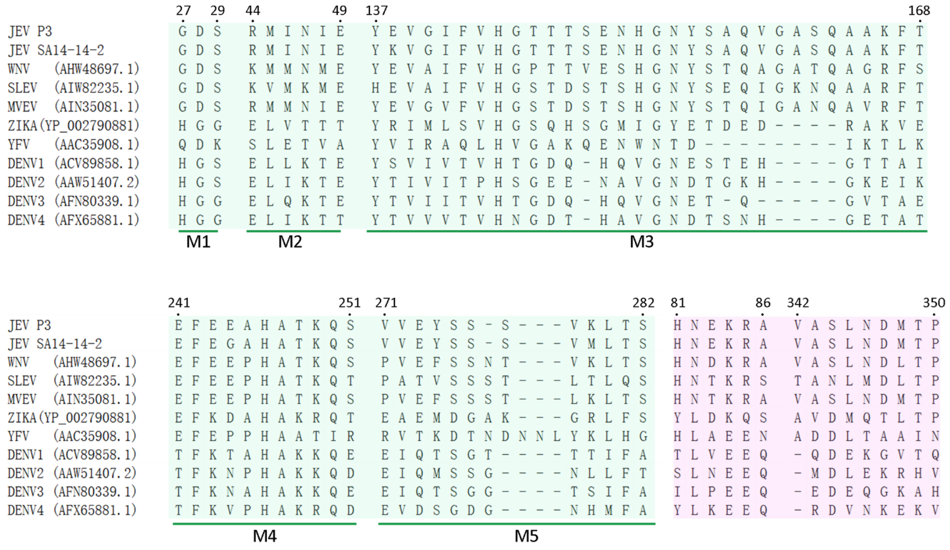

Supplementary Figure 8. Structure-based multiple sequences alignment

Structure-based amino acid sequence alignment of the E proteins from seven representative flaviviruses using Clustal X[8]. Seven encephalitic-specific motifs were mapped, five of which that locate on the external surface around the "holes" are highlighted in green and named M1, M2, M3, M4 and M5, respectively. The remaining two motifs are highlighted in pink.

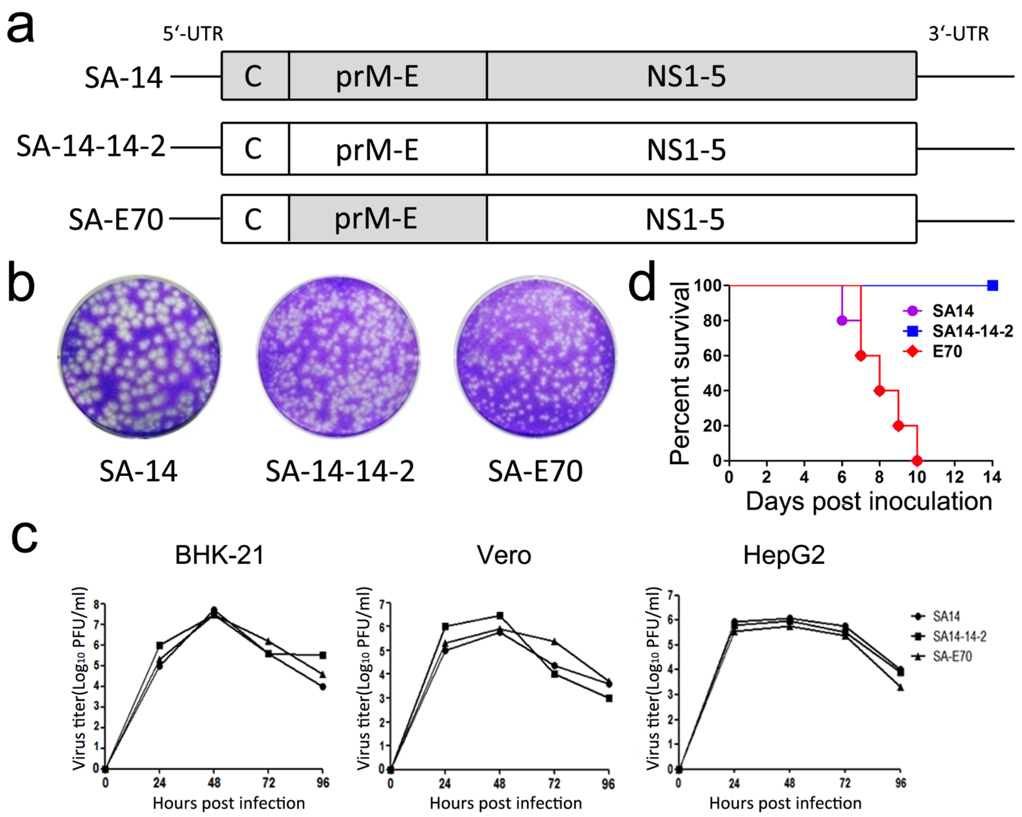

Supplementary Figure 9. The E protein predominantly determines the virulence in mice

(a) Schematic representation of the genomes of SA14, SA14-14-2 and the chimera SA-E70. **(b)** Plaque phenotypes of SA14, SA14-14-2 and the chimera SA-E70. **(c)** Growth curves of SA14, SA14-14-2 and SA-E70 in BHK-21, Vero and HepG2 cells. The culture supernatants were harvested at indicated time points to estimate the titer of progeny by plaque assays using BHK-21 cells. **(d)** Survival curves of 3-week-old BALB/c mice infected with the SA14 (n=5), SA14-14-2 (n=5) or SA-E70 (n=5), 107 PFU/mouse, IP route.

.

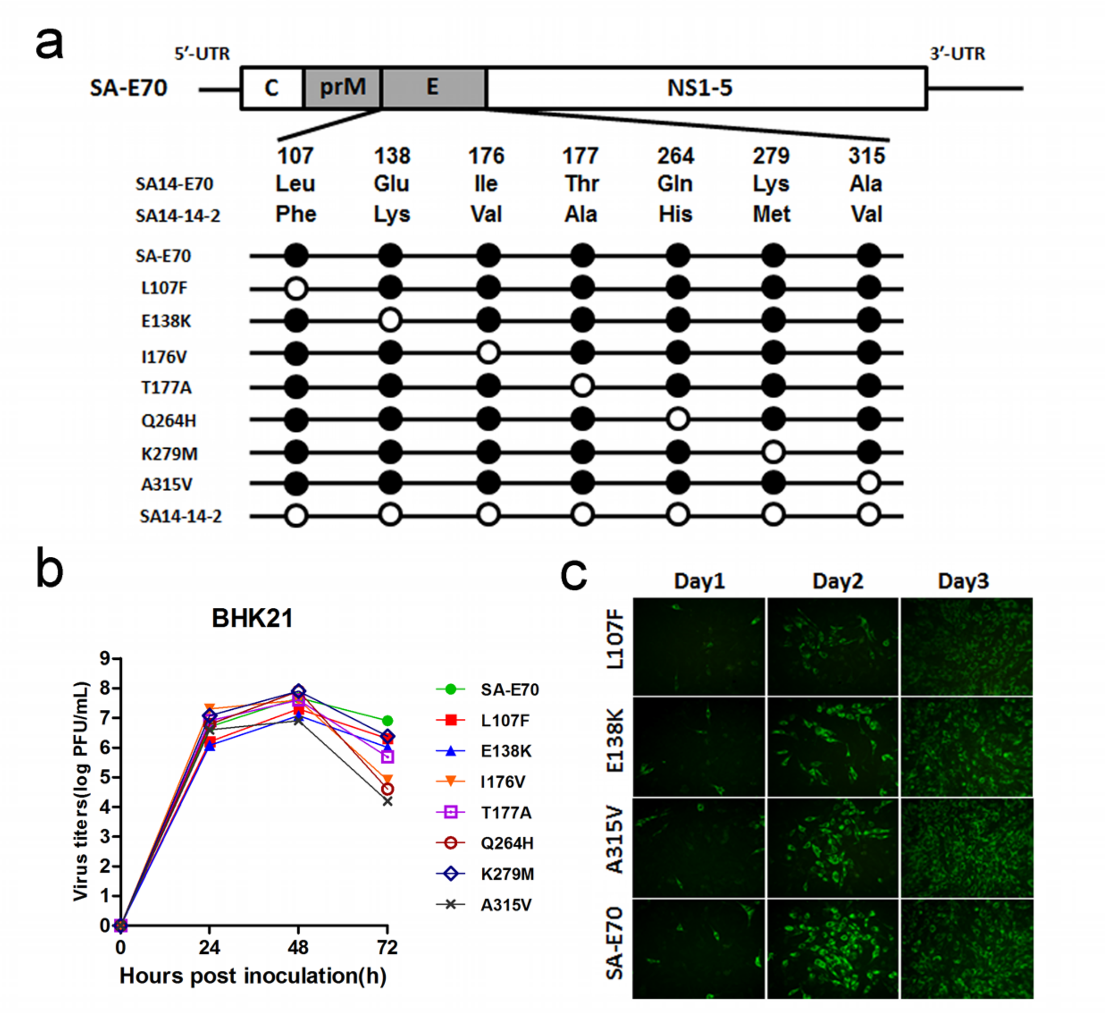

Supplementary Figure 10. Characterizations of the JEV mutant viruses.

(a) Graphical representation of the seven mutant viruses derived from SA-E70. **(b)** Growth properties of mutant viruses in BHK-21 cells. **(c)** Evaluation of E protein expression in JEV mutant viruses. BHK-21 cells infected with JEV mutant viruses were fixed at indicated time points post infection, and incubated with primary MAb 4D5, followed by incubation with secondary IgG antibodies conjugated to Alexa Fluor 488. Positive cells were detected using fluorescence microscopy.

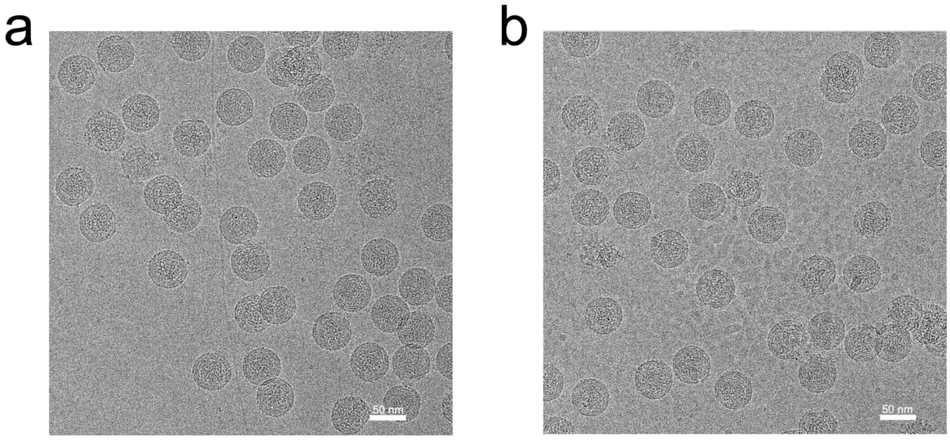

Supplementary Figure 11. Cryo-EM images of JEV virions (P3 strain) produced from Vero cells (Left) and C6/36 cells (Right) at 37°C.

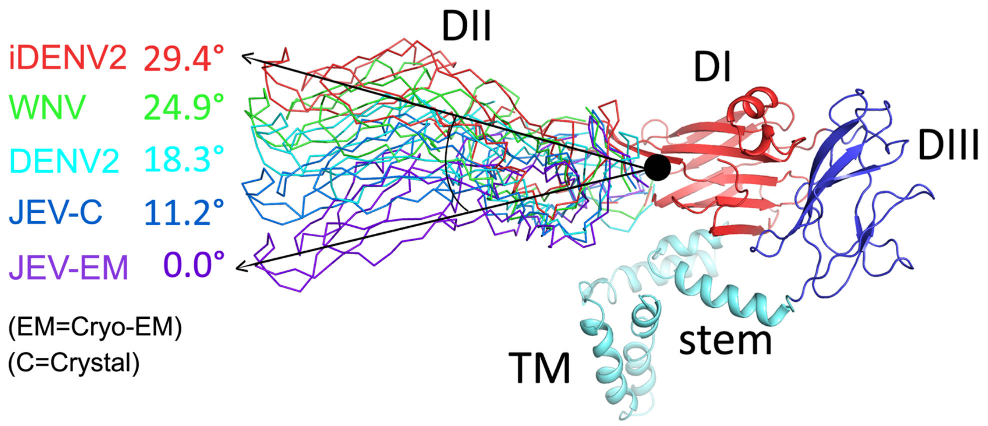

Supplementary Figure 12. Comparison of E protein DI and DII hinge angles.

JEV-E: cryo-EM structure of the JEV (P3 strain); JEV-C: crystal structure of solubilized ectodomain of JEV[4]; DENV2: cryo-EM structure of the DENV2[3]; WNV: crystal structure of solubilized E ectodomain of WNV[9]; iDENV2: crystal structure of immature E ectodomain of DENV2[10]. Domain II are colored according to the virus of origin and the numbers on the left indicate the difference in angle between DI and DII of each E protein and JEV-E E protein.

Supplementary Table 1. Cryo-EM imaging, data processing and models refinement statistics.

Name	JEV mature virus
Data collection	
Micrographs (total)	1,866
Micrographs (used)	1,426
Particles selected	30,558
Particles included in final reconstruction	15,035
Sampling, Å per pixel	1.35
Defocus range, µm	1.0-2.5
Resolution (Å)	4.3
(FSC = 0.143 criterion)	
Models Refinement	
Ramachandran statistics (%)	
Most favored	90.23
Allowed	7.95
Outliers	1.81
R.m.s.deviations	
Bond lengths(Å)	0.008
Bond angles(°)	1.322
Rwork/Rfree (%)	36.2/36.5

Supplementary Table 2. Amino acid residues that are different in the E protein among JEV P3, JEV SA14 and JEV SA14-14-2 strains

Amino acid position	Amino acid in the E protein in JEV strain		
	P3	JEV SA14	SA14-14-2
76	Met	Thr	Thr
107	Leu	Leu	Phe
138	Glu	Glu	Lys
176	Ile	Ile	Val
177	Thr	Thr	Ala
227	Pro	Ser	Ser
244	Glu	Gly	Gly
264	Gln	Gln	His
279	Lys	Lys	Met
306	Gly	Glu	Glu
315	Ala	Ala	Val
408	Leu	Ser	Ser
439	Lys	Lys	Arg
