# Supplementary material for: Near-atomic structure of Japanese encephalitis virus reveals critical determinants of virulence and stability
Source: Nat Commun. 2017 Apr 26;8:14. doi: 10.1038/s41467-017-00024-6 (PMC5432033; doi:10.1038/s41467-017-00024-6)
Supplement: Supplementary file 2 — Reviewer reports and authors' response from the peer review of this Article at Nature Communications [file 41467_2017_24_MOESM2_ESM.pdf]

PEER REVIEW FILE

Reviewers' Comments:

Reviewer #1 (Remarks to the Author):

This paper describes the cryo-EM structure of the japanese encephalitis virus (JEV) at 4.3Å resolution. This is an important structure, providing insight into the stabilization of the head-to-tail dimer conformation of the E protein at the surface of the virion, in contrast of the soluble E ectodomain (sE), which behaves as monomer in solution. The structure shows that there are a number of ionic interactions between the base of the ectodomain and the helices of the stem region, which lie flat on the membrane and keep the E dimer flat on the surface, stabilizing the dimeric conformation. They also observe a number of interactions between the ectodomain of M and the E dimer that also contribute to the pre-fusion conformation.

Although this is the third structure of a flavivirus particle at this resolution (after those of dengue virus and of zika virus), it is important because it allows for a number of interesting and insightful comparisons. As flaviviruses are important emerging or re-emerging viruses, which can cause explosive epidemics around the word, as illustrated by the current Zika virus outbreak in South America and in the Caribbean, the details for the overall organization of one of them presented here has a high potential impact. I therefore think that this is an important result but I didn't find the manuscript very clear and not all the claims justified. The authors should revise the manuscript as indicated below for further consideration by the journal.

Major points:

1- The paper is presented as providing insight into the neurotropism of JEV. It is not very clear to me what additional insight can be extracted from mapping the residues that are important for neurovirulence onto the E dimer in this structure than could not be done on the available structure of the sE protein from JEV, which in spite of being monomeric in solution makes a head-to-tail dimer in the crystals. That sE dimer also presents holes at the interface, which the authors claim to have discovered here.

2- Because the most interesting part is the comparative study, it would be important to move supplementary Figure 2 into a main Figure. The value of this Figure is to present the three viruses at the same scale, showing that the JEV particle is slightly larger than the others. Also, it is important to highlight in that same Figure the "holes", and also point to the corresponding location on ZIKV and DENV2.

3- What new insight is providing Figure 3C? This panel is some sort of "deja vu" (see Zhang et al, Structure 2004 12:1607-1618)

4- Fig. 5 shows that mutant E138K is already somewhat crippled since it has a small-plaque phenotype. It is attenuated in the mice even when it is introduced intra-cerebrally. The author should use as control another non-neuroinvasive viruses to be able to conclude something about this type of inoculation, or else it seems that the mutation simply cripples the virus which become attenuated, independent of what cells it infects. In contrast, A315V and L107F do not appear crippled in the plaque assay, are as neurovirulent as the wild-type when introduced i.c., but have reduced neuroinvasiveness, according to Figure 5C. It seems to me that this is an interesting finding, which the authors seem to overlook, and concentrate essentially on the E138K mutant, which there data suggest is not as interesting.

In summary, the title of the manuscript is misleading and should be changed. The data presented does not support the claim that the structure of JEV reveals the basis of flavivirus neurotropism.

Minor Points

1- The sentence in page 3: "Worldwide, approximately 50,000-175,000 clinical cases of JE are reported in more than 25 countries annually5, with JEV being the most common agent.". What does it mean? That JEV is the most common agent of JE?

2- The manuscript lacks something like and Espript file with the amino acid sequence and the secondary and tertiary structure indicated and labeled on top as well as on a carton of the dimer. It is very difficult otherwise to the reader to know where is helix αB, for instance, which is mentioned many times on the manuscript, as well as the other strands, loops, etc.

3- It would be important to show how the RGD residues are exposed on the virion, it is only mentioned that they are exposed about the 3- and 5-fold axes, but they are only sown in Fig. 1C and D in the context of an isolated E dimer. It would be important to show them displayed as in the current Supplementary Fig. 2A, but zooming on the 3-fold and 5-fold axis to show these elements.

4- The stretch of residues from 279- 297 appear to map to strand I0 and the linker between domain I and III? If so, please say so.

5- At the top of page 11, the authors discuss histidines and pH sensors. And they forget to cite one of the most important results on this issue, by Zheng et al, Nat. Commun. 2014, 5:3877. doi: 10.1038/ncomms4877.

Reviewer #2 (Remarks to the Author):

Wang et al. have solved the cryo-EM structure of JEV - a valuable addition to the field of flavivirology. The authors have made a number of important observations, including details of which amino acids contribute to neurovirulence, particularly E138, and the pH stability of JEV. The experimental work is of high quality, this is an important contribution to the field, and

should be published. However, the title of the paper is overstated. As the receptor(s) for JEV on neuronal cells are not yet identified, the title claims more than can be supported by the data, although the authors have certainly contributed a lot to our current understanding of neurotropism.

The major problem with the work is that the structure of P3 has been determined, but most of the experimental work has used SA14/SA14-14-2, making direct comparison with P3 a little problematic. For example, the authors show that the E138K mutation is crucial in determining neurovirulence. However, as shown in the paper of Yun et al, (PLOS Pathog 2014) a virus containing this mutation can still be virulent, by introducing a Gly to Glu change at position 244. This is a reversion to make SA14-14-2 more like P3 - SA14 also contains Gly at this position, which may be one reason why it is relatively attenuated compared with P3. This should be discussed in more detail.

The authors provide evidence that a Q264H change results in greater stability of SA14-14-2 to temperature and pH. However, these experiments should be extended to include full length SA14, and P3. Otherwise, it is hard to interpret the role of pH stability in attenuation of the virus, especially as the authors have shown that this mutation alone does not confer attenuation.

The following are discussion points which should be included/addessed:

1. The virus studied was grown in Vero cells - however there is evidence that growing JEV in different cell types may affect the glycosylation of the envelope protein (Shimojima, M., A. Takenouchi, et al. (2014). "Distinct usage of three C-type lectins by Japanese encephalitis virus: DC-SIGN, DC-SIGNR, and LSECtin." Arch Virol 159(8): 2023-2031). There is no mention that this structure might be different if the virus was derived from a different cell type, in particular if derived from insect cells, which be more reflective of what a host is infected with in nature.

2. The statement is made that the “site of attachment for neuronal receptors will presumably be conserved” (lines 235-7). The authors go on to point out that many of the crucial residues that are conserved in encephalitic flaviviruses are the opposite charge in DENV. Yet, DENV can get into the brain and cause encephalitis, as can all flaviviruses that cause significant disease in man (even yellow fever vaccine, rarely). Presumably, therefore, there must be more to this than just the difference between the predominantly encephalitic versus predominantly non-encephalitic viruses?

3. The authors may have shown a role for the fusion loop in crossing the blood brain barrier - but this is not discussed further.

4. Line 146: However Zika seems to be more phylogenetically similar to dengue based on linear sequence (see the Screaton group Nat Immunol paper on enhancement of Zika by dengue antibodies). Could the authors discuss this difference?

A number of other more minor points are:

Introduction lines 56 - 82: this could be shortened to allow further discussion of the above if needed.

Results line 102 - 115: The authors have worked on three strains of JEV - it should be clearly stated that the structure shown is that of JEV P3 whenever the data are directly referred to. The figure legend should also state that it is the structure of P3 that is presented.

Suppl. Fig 3:
3b - state that EM = cryo-EM and C = crystal.
3c - this will be much easier to read if the colour scheme is given rather than referring to another figure legend. The scheme should simply be given again in the Fig 3c legend.

Lines 152-4: This was hinted at by Eiji Konishi in the early 90s: Konishi, E. and P. W. Mason (1993). "Proper maturation of the Japanese encephalitis virus envelope glycoprotein requires cosynthesis with the premembrane protein." J Virol 67(3): 1672-1675. This could be cited.

Supplementary information - mouse experiments - I think one “neurovirulence” should read “neuroinvasiveness” in this section.

Some additional minor points are on the attached word document.

Manuscript NCOMMS-16-15656-T

Response to referees' comments

We thank the reviewers for their positive and constructive comments, and now we have performed additional experiments, re-organized several figures, polished the text throughout the manuscript, and responded point-by-point to the reviewers' comments. We believe we have clarified all the reviewers' concerns and produced a more balanced story.

Reviewer #1 (Remarks to the Author):

This paper describes the cryo-EM structure of the Japanese encephalitis virus (JEV) at 4.3Å resolution. This is an important structure, providing insight into the stabilization of the head-to-tail dimer conformation of the E protein at the surface of the virion, in contrast of the soluble E ectodomain (sE), which behaves as monomer in solution. The structure shows that there are a number of ionic interactions between the base of the ectodomain and the helices of the stem region, which lie flat on the membrane and keep the E dimer flat on the surface, stabilizing the dimeric conformation. They also observe a number of interactions between the ectodomain of M and the E dimer that also contribute to the pre-fusion conformation.

Although this is the third structure of a flavivirus particle at this resolution (after those of dengue virus and of zika virus), it is important because it allows for a number of interesting and insightful comparisons. As flaviviruses are important emerging or re-emerging viruses, which can cause explosive epidemics around the word, as illustrated by the current Zika virus outbreak in South America and in the Caribbean, the details for the overall organization of one of them presented here has a high potential impact. I therefore think that this is an important result but I didn't find the manuscript very clear and not all the claims justified. The authors should revise the manuscript as indicated below for further consideration by the journal.

Major points:
1. The paper is presented as providing insight into the neurotropism of JEV. It is not very clear to me what additional insight can be extracted from mapping the residues that are important for neurovirulence onto the E dimer in this structure than could not be done on the available structure of the sE protein from JEV, which in spite of being monomeric in solution makes a head-to-tail dimer in the crystals. That sE dimer also presents holes at the interface, which the authors claim to have discovered here.
Response:
Drawing inferences about an assembled particle from isolated structures is possible but is inevitably speculative, however having the structure of the assembled shell that we present gives great confidence in these observations. We have acknowledged the congruence of the structures of the isolated sE protein.

2. Because the most interesting part is the comparative study, it would be important to move supplementary Figure 2 into a main Figure. The value of this Figure is to present the three viruses at the same scale, showing that the JEV particle is slightly larger than the others. Also, it is important to highlight in that same Figure the "holes", and also point to the corresponding location on ZIKV and DENV2.
Response:

Thanks. Supplementary Figure 2 has been modified as suggesting and moved to become a main figure (Figure 2)

3. What new insight is providing Figure 3C? This panel is some sort of "deja vu" (see Zhang et al, Structure 2004 12:1607-1618)

Response:

Thanks. We agree. We have moved Figure 3C into a supplementary figure and cited the mentioned paper.

4. Fig. 5 shows that mutant E138K is already somewhat crippled since it has a small-plaque phenotype. It is attenuated in the mice even when it is introduced intra-cerebrally. The author should use as control another non-neuroinvasive viruses to be able to conclude something about this type of inoculation, or else it seems that the mutation simply cripples the virus which become attenuated, independent of what cells it infects. In contrast, A315V and L107F do not appear crippled in the plaque assay, are as neurovirulent as the wild-type when introduced i.c., but have reduced neuroinvasiveness, according to Figure 5C. It seems to me that this is an interesting finding, which the authors seem to overlook, and concentrate essentially on the E138K mutant, which there data suggest is not as interesting.

Response:

Thanks for the comments. Whilst E138K showed small plaque phenotype, its growth and viral protein expression in BHK-21 cells remained the same as WT and other mutants (this was shown in the original supplemental Fig. 9). Thus, the observed phenotype in mice is directly associated with neurovirulence as determined using the well-recognised intracerebrally injection approach. Of course, A315V and L107F are also importance for neuroinvasiveness, however, the role of these two critical amino acid residues are not directly linked to our structural findings. Thus, we were not able to dissect the detailed mechanism in our present manuscript.

In summary, the title of the manuscript is misleading and should be changed. The data presented does not support the claim that the structure of JEV reveals the basis of flavivirus neurotropism.

Response:

We have changed the title to: "Near-atomic structure of Japanese encephalitis virus reveals critical determinants of virulence and stability"

Minor Points

1. The sentence in page 3: "Worldwide, approximately 50,000-175,000 clinical cases of JE are reported in more than 25 countries annually5, with JEV being the most common agent.". What does it mean? That JEV is the most common agent of JE?

Response:

Thanks. We have corrected this sentence as to read "Worldwide, approximately 50,000-175,000 clinical cases of Japanese encephalitis are reported in more than 25 countries annually."

2. The manuscript lacks something like an Espript file with the amino acid sequence and the secondary and tertiary structure indicated and labeled on top as well as on a carton of the dimer. It is very difficult otherwise to the reader to know where is helix αB, for instance, which is mentioned many times on the manuscript, as well as the other strands, loops, etc.

Response:

Thanks. This has been added.

3. It would be important to show how the RGD residues are exposed on the virion, it is only mentioned that they are exposed about the 3- and 5-fold axes, but they are only sown in Fig. 1C and D in the context of an isolated E dimer. It would be important to show them displayed as in the current Supplementary Fig. 2A, but zooming on the 3-fold and 5-fold axis to show these elements.
Response:
Thanks. This has been added in Figure 1C.

4. The stretch of residues from 279- 297 appear to map to strand I0 and the linker between domain I and III? If so, please say so.
Response:
Thanks. This has been clarified (Line 131).

5. At the top of page 11, the authors discuss histidines and pH sensors. And they forget to cite one of the most important results on this issue, by Zheng et al, Nat. Commun. 2014, 5:3877. doi: 10.1038/ncomms4877.
Response:
Thanks. This has been done.

Reviewer #2 (Remarks to the Author):

Wang et al. have solved the cryo-EM structure of JEV - a valuable addition to the field of flavivirology. The authors have made a number of important observations, including details of which amino acids contribute to neurovirulence, particularly E138, and the pH stability of JEV. The experimental work is of high quality, this is an important contribution to the field, and should be published. However, the title of the paper is overstated. As the receptor(s) for JEV on neuronal cells are not yet identified, the title claims more than can be supported by the data, although the authors have certainly contributed a lot to our current understanding of neurotropism.

The major problem with the work is that the structure of P3 has been determined, but most of the experimental work has used SA14/SA14-14-2, making direct comparison with P3 a little problematic. For example, the authors show that the E138K mutation is crucial in determining neurovirulence. However, as shown in the paper of Yun et al, (PLOS Pathog 2014) a virus containing this mutation can still be virulent, by introducing a Gly to Glu change at position 244. This is a reversion to make SA14-14-2 more like P3 - SA14 also contains Gly at this position, which may be one reason why it is relatively attenuated compared with P3. This should be discussed in more detail.
Response:
Thanks. We agree indeed that the residue Glu244 also important for neurovirulence. Additional adaptive mutations or reversion may occur during various culturing or passaging history, indeed some other residues that may affect viral neurovirulence cannot be be fully identified from structural and reverse genetic studies. We have now cited the 2014 PLoS Pathogens paper and discussed the importance of position of 244 in the revised manuscript.

The authors provide evidence that a Q264H change results in greater stability of SA14-14-2 to temperature and pH. However, these experiments should be extended to include full length SA14, and P3. Otherwise, it is hard to interpret the role of pH stability in attenuation of the

virus, especially as the authors have shown that this mutation alone does not confer attenuation.

Response:

As shown in Fig 5, our stability comparison was based on reverse genetic approach, and there is only a single amino acid mutation Q264H between the two viruses. Also, we have now compared the stability of the three wild type viruses (SA14, SA14-14-2 and P3), and the results showed that SA14-14-2 (containing H264 at E protein) was more stable than either SA14 and P3 (containing Q264 at E protein), in agreement with our conclusion from reverse genetic experiments. We have now included these findings in a new Supplemental Fig 6 and revised the main text accordingly.

The following are discussion points which should be included/addressed:

1. The virus studied was grown in Vero cells - however there is evidence that growing JEV in different cell types may affect the glycosylation of the envelope protein (Shimojima, M., A. Takenouchi, et al. (2014). "Distinct usage of three C-type lectins by Japanese encephalitis virus: DC-SIGN, DC-SIGNR, and LSECtin." Arch Virol 159(8): 2023-2031). There is no mention that this structure might be different if the virus was derived from a different cell type, in particular if derived from insect cells, which be more reflective of what a host is infected with in nature.

Response:

Thanks. We discussed this issue in the discussion section (Line 316 onwards), in this sections we also make clear that we did not observe notable structural changes between JEV produced from vero and insect cells at 37 ° C, both particles show a smooth appearance.

2. The statement is made that the "site of attachment for neuronal receptors will presumably be conserved" (lines 235-7). The authors go on to point out that many of the crucial residues that are conserved in encephalitic flaviviruses are the opposite charge in DENV. Yet, DENV can get into the brain and cause encephalitis, as can all flaviviruses that cause significant disease in man (even yellow fever vaccine, rarely). Presumably, therefore, there must be more to this than just the difference between the predominantly encephalitic versus predominantly non-encephalitic viruses?

Response:

Thanks. Indeed our structure-based sequences alignment of representative flaviviruses provides clues to the common characteristics of the Japanese encephalitis group and to discover the differences with other groups, which is useful for us in designing experimental assays to verify our speculations. We have therefore corrected the classifications of encephalitic, non-encephalitic viruses to be JEV group.

3. The authors may have shown a role for the fusion loop in crossing the blood brain barrier - but this is not discussed further.

Response:

Sorry, we did not intend to discuss the role for the fusion loop in crossing the blood brain barrier in this manuscript. If we have implied this we will correct.

4. Line 146: However Zika seems to be more phylogenetically similar to dengue based on linear sequence (see the Screaton group Nat Immunol paper on enhancement of Zika by dengue antibodies). Could the authors discuss this difference?

Response:

Thanks. Results can vary a little between different approaches. Regarding to which approach

(Structure-based or Sequence-based evolution analysis) is better, one paper (we have cited) summarized these two methods and suggests that structure-based phylogeny is more reliable if the sequence similarities are relatively low. Whether ZIKV is more close phylogenetically to DENV or JEV, we all believe that ZIKV links DENV and JEV and shares similarities from both. Although the Nat Immunol paper reported infection of ZIKV could be enhanced by dengue antibodies, we don't know if can it also be enhanced by JEV antibodies? This is beyond the scope of our paper.

A number of other more minor points are:

Introduction lines 56 - 82: this could be shortened to allow further discussion of the above if needed.
Response:
Thanks for suggestions. We have another manuscript (in preparation) that analyzes the evolution of ZIKV. We focus on the structural determinants for JEV neurovirulence and stability in this manuscript.

Results line 102 - 115: The authors have worked on three strains of JEV - it should be clearly stated that the structure shown is that of JEV P3 whenever the data are directly referred to. The figure legend should also state that it is the structure of P3 that is presented.
Response:
Thanks. This has been done.

Suppl. Fig 3:
3b - state that EM = cryo-EM and C = crystal.
3c - this will be much easier to read if the colour scheme is given rather than referring to another figure legend. The scheme should simply be given again in the Fig 3c legend.
Response:
Thanks. These changes have been made.

Lines 152-4: This was hinted at by Eiji Konishi in the early 90s: Konishi, E. and P. W. Mason (1993). "Proper maturation of the Japanese encephalitis virus envelope glycoprotein requires cosynthesis with the premembrane protein." J Virol 67(3): 1672-1675. This could be cited.
Response:
Thanks. This has been done.

Supplementary information - mouse experiments - I think one "neurovirulence" should read "neuroinvasiveness" in this section.
Response:
Thanks. This has been done.

Some additional minor points are on the attached word document.
Response:
Thanks. This has been done.

<u>Reviewers' Comments:</u>

Reviewer #1 (Remarks to the Author):

This revised version is much better than the initial version the authors had. The authors have addressed all the changes required. The title reflects better the findings, and the new Figures have made the description much more clear. It is a very important advance to have the cryo-EM structure to such details of a third member of the flavivirus genus, and the accompanying comparative analysis is extremely informative. I strongly recommend publication of this version of the manuscript.

Reviewer #2 (Remarks to the Author):

Second review of 101372 Wang et al JEV virion structure.

My comments have mostly been addressed. However, there are a number of minor modifications that must made before I can recommend publication of the paper. In particular, Suppl figs line 105 – 114 – C and EM are still not defined in the legend to Suppl fig 4 (prev Suppl fig 3), and the colour scheme is not given. The authors state in the rebuttal they have made the changes, yet I do not see them, the new Suppl fig 4 legend appears identical to the prev Suppl fig 3 legend, apart from the fig number. Both of these are minor changes and add would add clarity.

There are a number of other changes that need to be made:

Line 50 – "encephalitis" – no capital

Line 85 – it would be better to say: "understanding of the pathogenesis of encephalitic flaviviruses in the JE sero-group" if that is what is meant – as it stands the sentence is clear to someone who works on flaviviruses, but may not be so clear to someone not familiar with flavivirus taxonomy/classification. The paper has much general relevance/importance – this will make it more accessible to those outside the field.

Line 250 – refers to suppl fig 7 when I think fig 8 is meant. (And double check that the correct suppl figs are referred to throughout.)

Line 254-255 – "this region of the surface exhibits distinct electrostatic surface between…" Is this right? Should it be exhibits distinct electrostatic characteristics? Or "this region exhibits a distinct electrostatic surface… "

Line 341 – remove "we"

Manuscript NCOMMS-16-15656-A

Response to referees' comments

We thank the reviewers for their second review. The final few points have been addressed.

REVIEWERS' COMMENTS:

Reviewer #1 (Remarks to the Author):

This revised version is much better than the initial version the authors had. The authors have addressed all the changes required. The title reflects better the findings, and the new Figures have made the description much more clear. It is a very important advance to have the cryo-EM structure to such details of a third member of the flavivirus genus, and the accompanying comparative analysis is extremely informative. I strongly recommend publication of this version of the manuscript.

Reviewer #2 (Remarks to the Author):

Second review of 101372 Wang et al JEV virion structure.

My comments have mostly been addressed. However, there are a number of minor modifications that must made before I can recommend publication of the paper. In particular, Suppl figs line 105 – 114 – C and EM are still not defined in the legend to Suppl fig 4 (prev Suppl fig 3), and the colour scheme is not given. The authors state in the rebuttal they have made the changes, yet I do not see them, the new Suppl fig 4 legend appears identical to the prev Suppl fig 3 legend, apart from the fig number. Both of these are minor changes and add would add clarity.

The legend to Supp. Fig .4 has been clarified to define C and EM and the colour scheme for parts a) and b) (given by a key in the figure) stated and the colour scheme for part c) corrected to reflect that it matches that in Fig. 1d.

There are a number of other changes that need to be made:

Line 50 – "encephalitis" – no capital

Corrected.

Line 85 – it would be better to say: "understanding of the pathogenesis of encephalitic flaviviruses in the JE sero-group" if that is what is meant – as it stands the sentence is clear to someone who works on flaviviruses, but may not be so clear to someone not familiar with flavivirus taxonomy/classification. The paper has much general relevance/importance – this will make it more accessible to those outside the field.

Thank you we have adopted this suggestion.

Line 250 – refers to suppl fig 7 when I think fig 8 is meant. (And double check that the correct suppl figs are referred to throughout.)

Yes, this has been corrected and the figure call outs re-checked.

Line 254-255 – “this region of the surface exhibits distinct electrostatic surface between…” Is this right? Should it be exhibits distinct electrostatic characteristics? Or “this region exhibits a distinct electrostatic surface… “

Thanks - we have adopted the latter suggestion.

Line 341 – remove “we”

Done.
